# Dynamic Properties of Network Motifs Contribute to Biological Network Organization

**DOI:** 10.1371/journal.pbio.0030343

**Published:** 2005-10-04

**Authors:** Robert J Prill, Pablo A Iglesias, Andre Levchenko

**Affiliations:** **1**Department of Biomedical Engineering, Johns Hopkins University, Baltimore, Maryland, United States of America,; **2**Department of Electrical and Computer Engineering, Johns Hopkins University, Baltimore, Maryland, United States of America; University of California, IrvineUnited States of America

## Abstract

Biological networks, such as those describing gene regulation, signal transduction, and neural synapses, are representations of large-scale dynamic systems. Discovery of organizing principles of biological networks can be enhanced by embracing the notion that there is a deep interplay between network structure and system dynamics. Recently, many structural characteristics of these non-random networks have been identified, but dynamical implications of the features have not been explored comprehensively. We demonstrate by exhaustive computational analysis that a dynamical property—stability or robustness to small perturbations—is highly correlated with the relative abundance of small subnetworks (network motifs) in several previously determined biological networks. We propose that robust dynamical stability is an influential property that can determine the non-random structure of biological networks.

## Abstract 2

The authors model how network motifs respond to small-scale perturbations and find a strong correlation between motif stability and abundance in a network, suggesting that dynamic properties of network motifs may play a role in overall network structure.

## Introduction

Life is a dynamical process. As the intricate connectedness of biological systems is revealed, it is essential to keep in mind that network maps are graphical representations of dynamic systems. A network with fixed structure is dynamic in the sense that the nodes take on values corresponding to *activities* that change in time. In a specific context, these activities may represent the concentration of a molecule, phosphorylation state of an enzyme, depolarization state of a neuron, etc. For example, the neural connection map of the nematode, *Caenorhabditis elegans*, is fixed in the adult worm, and invariant from individual to individual [1]. Although the network structure is static, the behavior of the neural network is dynamic. At the lowest level, the dynamic nature of the system is exhibited as depolarizations of individual neurons. At an intermediate level of organization, each neuron potentially influences the behavior of its nearest neighbors via synaptic connections.

Example biological networks, such as those analyzed here, are artificially separated from the highly integrated and complex whole, which includes interconnected metabolic, signal transduction, transcriptional, cytoskeletal, and other types of interlocked networks and pathways. Even with this limitation, consideration of the structure of isolated networks has profoundly influenced contemporary biology, which is increasingly focused on systems-level concepts. The topological structures of the networks studied here: the transcriptional regulatory networks of *Escherichia coli, Saccharomyces cerevisiae,* the developmental transcriptional network of *Drosophila melanogaster,* the signal transduction knowledge environment (STKE) network, and the neural connection map of *C. elegans,* have been analyzed from various perspectives, leading to provocative ideas relating structure to function. These and other natural networks have non-Poisson degree distribution (often power-law) [2]. In addition, recent studies have indicated that certain patterns of local connectivity (network motifs) are statistically over- and underrepresented in various networks, including those regulating development and function of living organisms [3,4]. The term “network motif” refers to a directed subgraph, consisting of a few nodes, that is embedded in a larger directed graph. Some motifs, such as the three-node feed-forward loop, may perform specific regulatory functions [5–7], although in general it is not presumed that motif instances are necessarily functional modules. Used as a structural analysis technique, enumeration of all subgraphs consisting of three or four nodes summarizes the local connectivity patterns that compose a complex network [8–10].

Presently, it is not clear what determines the particular frequencies of all possible network motifs in a specific network. At least two alternative explanations can be offered. One can hypothesize that certain constraints on development of a network as a whole determine which motifs become abundant. Conversely, some network motifs may possess properties important enough to become overrepresented and thereby drive the network evolution and its ultimate structure. The latter explanation is consistent with the frequently made assumption that the function of a small biological network or pathway is largely determined by the connections among the constituting genes, proteins, metabolites, or cells [11–16]. Many of the genetic and biochemical systems extensively studied to date as functional units, such as the Lac and Che systems in *E. coli* (responsible for lactose utilization and chemotaxis, respectively) are of this sort [17–19]. In these systems, connectivity is typically translated into a dynamic property, which, in turn, takes on a functional meaning. For instance, a positive feedback loop in the Lac system (a structural characteristic) is used to implement a biochemical switch (dynamic behavior) that acts as a primitive memory mechanism (functional role). We hypothesize that the dynamic behavior displayed by a network motif is an important criterion in determination of functional significance of the motif and, potentially, its abundance in biological networks. Further, we propose that it is computationally tractable to determine certain dynamic properties for every possible network motif. This strategy can explain why certain network motifs are overrepresented in real biological networks while others are not.

A comprehensive analysis of the dynamics of networks, large or small, is considerably more complicated than the corresponding analysis of their structure. For instance, structures of all possible three- or four-node network motifs can be generated and enumerated easily. However, their dynamics may not be completely determined owing to unknown and potentially complex functional dependencies between nodes, the lack of knowledge of parameters defining specific instances of motifs in real networks, and “unmodeled” interactions that may be absent from the network representation yet relevant to dynamics. In addition, motifs with the same topology may give rise to different dynamic behaviors, as was recently demonstrated experimentally using small synthetic genetic circuits [20]. Clearly, there is not a direct one-to-one relationship between network structure and possibly complex system dynamics. However, one can try to address these fundamental challenges by taking advantage of the widely acknowledged notion that biological systems perform various functions robustly, i.e., under wide ranges of their parameters [21]. Thus, instead of considering properties of a particular instance of a motif, we analyze generic properties that arise from the topology of the network motif.

Although the particular parametric values can confer unique properties onto individual instances of motifs, we argue that all instances of a particular motif display characteristics that can be studied comprehensively. Here we present the analysis of a specific robust property that can be displayed by network motifs: robust stability to small-scale perturbations of the activities of the biological entities. Small perturbations include intrinsic stochastic fluctuations (noise) and transient up- or down-regulation of activity. “Small” implies that a linear approximation of the potentially nonlinear relations is still valid (see Materials and Methods).

Intuitively, one expects that for complex biological networks to function robustly, it is necessary that they display stability and resist both noise and stressful small-scale perturbations. These basic homeostatic properties, however, are not inherent to any biological or biochemical system, as small stimuli can, in principle, trigger large-scale sustained responses, especially if feedback interactions between members of a biological network are involved. For example, relatively short stimulations by tumor necrosis factor α (5 min or less) can trigger sustained (60 min) activation of the NF-κB pathway leading to expression of a battery of genes [22]. Here we show that stability to small perturbations displayed robustly by network motifs can be characterized comprehensively. In addition, and more significantly, we show that this property can be a driving force defining the structure of several biological networks.

## Results

**Figure 1:**
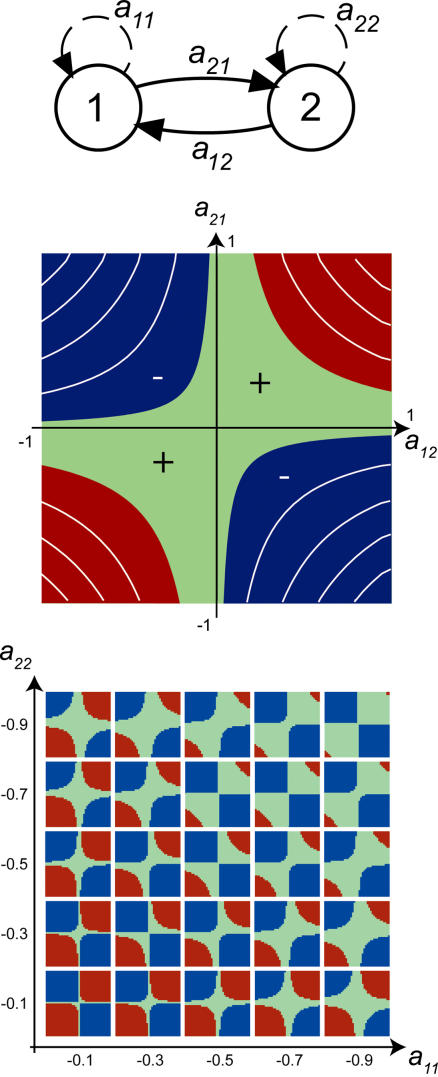
Dynamic Behaviors of a Two-Node Feedback Loop in Response to a Small Perturbation from Steady-State (Top) Schematic of a simple feedback circuit with the nodes and edges labeled. (Middle) We illustrate the dependence of stability on the values of *a*_12_ and *a*_21_ as they are varied from −1 to 1, with constant self-degradation terms *a*_11_ = −0.3 and *a*_22_ = −0.7. The system can be stable (green), oscillatory (blue), or unstable (red). These regions are determined by gain k = *a*_12_ *a*_21_. Shown in white are contours of constant gain k. The quadrants labeled (+) correspond to positive feedback (*a*_12_ and *a*_11_ have the same sign), while the quadrants labeled (−) correspond to negative feedback (*a*_11_ and *a*_21_ have opposite signs). (Bottom) The stability regions vary as the values of self-degradation terms *a*_11_ and *a*_12_ change. The more stable the open-loop nodes (more negative *a*_11_ and *a*_22_), the greater the regions of closed-loop stability. However, if *a*_11_ and *a*_22_ are close in sign and magnitude, the size of the oscillatory regions increases.

The dynamic behavior of a circuit is determined by the direction, sign, and strength of the connections. To develop some intuition, consider the case of a two-node feedback loop (Figure 1). The sign and strength of a connection from node *j* to node *i* is labeled *a_ij_* . Additionally, we assume that a self-interaction, denoted *a_ii_*, represents the commonly observed mechanisms of constitutive degradation or inactivation of the biological entities. Without proper mathematical intuition, one could incorrectly assume that negative feedback ensures stability and positive feedback destroys stability. As we demonstrate next, the dynamics of this simple circuit are more complicated.

We analyze the response of this circuit to a small perturbation from a steady-state under different assumptions on the parameters (Figure 1). For a particular set of constant values for the self-degradation terms *(a*_11_ *, a*_22_ *),* the system can be stable (green), unstable (red), or oscillatory (blue), depending on the sign and strength of the feedback gain *(k* = *a*_12_ *a*_21_ *)*. However, these regions occupy different relative areas of parameter space depending on the particular values of the self-degradation terms. The more stable the individual nodes (more negative *a*_11_ *, a*_22_), the greater the regions of closed-loop stability. Consequently, positive feedback produces stability if constitutive degradation dominates the feedback gain. Both positive and negative feedback can produce oscillations in general (only negative feedback can produce oscillations in the two-node case). Finally, upstream input nodes and downstream output nodes can be added to the system without altering the stability, provided that the number or size of feedback loops is unchanged.

Similar analysis can be extended to all possible three- or four-node network motifs by defining a metric, the structural stability score (SSS), as the probability that the dynamical system corresponding to a given motif relaxes monotonically to steady-state following a small perturbation. An SSS value of 1 indicates that non-oscillatory relaxation to a steady-state (henceforth simply termed stability) is guaranteed by connectivity and does not depend on the specific parameter values that define the functional interactions, thus making the system *structurally* stable. SSS scores less than unity indicate the extent to which parameter values influence stability: the lower the score, the more precise the balance of connection signs and strengths necessary to achieve stability. For example, the stability of a small transcriptional network with a high SSS would be robust to fluctuations of protein expression levels, seemingly the most important source of variation of gene expression across bacterial cell populations [23]. This compact description of the relationship between network structure and system dynamics enables a comprehensive characterization of the stability of small circuits in real networks.

Determination of the SSS values (fully described in Materials and Methods) reveals that all topologically distinct three-node (13 total) and four-node (199 total) network motifs partitioned into three classes that display distinct stability regimes. In particular, the first class (I) comprised all the robustly stable motifs (SSS = 1) devoid of feedback loops, i.e., motifs that are directed acyclic graphs. The second class (II) consisted of moderately stable circuits (SSS ≈ 0.4) containing a single two-node feedback loop. These motifs can be guaranteed not to be unstable (but may have damped oscillations) provided that the feedback present is negative (see Figure 1). The third class (III) contained motifs with much lower SSS values (< 0.2), which were a mixture of more complicated circuits: multiple two-node loops, three- and four-node loops, nested multi-node loops, etc. Their stability cannot be guaranteed by specifying the sign of the feedback loops present. Generally, as the number or length of loops increases, the SSS decreases. Thus, highly connected motifs are generally the least stable.

**Figure 2:**
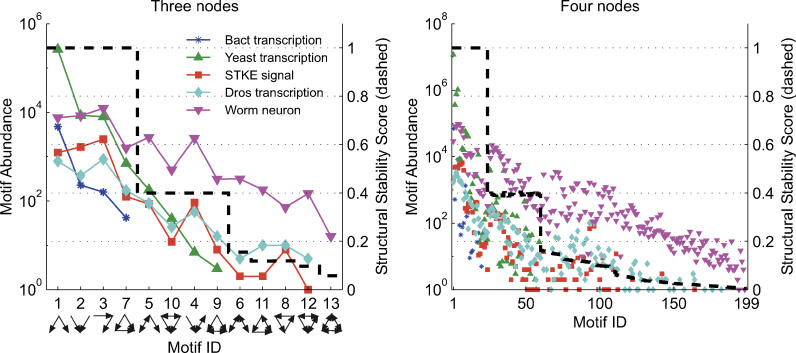
Abundance of a Motif Is Correlated with Its SSS The number of instances of all three-node (left) and four-node (right) motifs in biological networks (log scale), and SSS (black dashed line). The motifs are sorted on the *x*-axis from high to low SSS.

Next, we contrasted the abundance of motifs in several real biological networks with the corresponding motif SSS values (Figure 2). Remarkably, in all networks analyzed, the stability scores showed excellent correlation with the motif abundance. The networks are mostly composed of the motifs in the highest stability class, suggesting that the overall network behavior is stable to small perturbations. This finding further suggests that the overall network structure may be driven by the requirement of stability to small perturbations, such as noise.

Above, we chose a conservative definition of stability, classifying any system with oscillatory behavior as unstable, even if the oscillations are damped. However, our results still hold if we adopt the more traditional, and less conservative, definition of stability in which damped oscillations are classified as stable dynamics, irrespective of the potential presence of oscillation (see Figures S1 and S2, damped oscillations in the SSS metric). Thus, our conclusions do not have a strong dependence on the particular definition of stability used.

We also noted that, for the same SSS values, the motif abundance was correlated with the number of edges in the motif, as can be seen from the particular ordering of the three-node motifs used (Figure 2, left panel). Since all the networks considered here are very sparse (compared to fully connected graphs of the same number of nodes), it stands to reason that motifs containing a large number of edges might be encountered less frequently than motifs with a low number of edges, independently of the stability properties. In subsequent analyses we focused on a comparison of relative abundances of network motifs within motif groups having the same number of edges (a “density group”).

**Figure 3:**
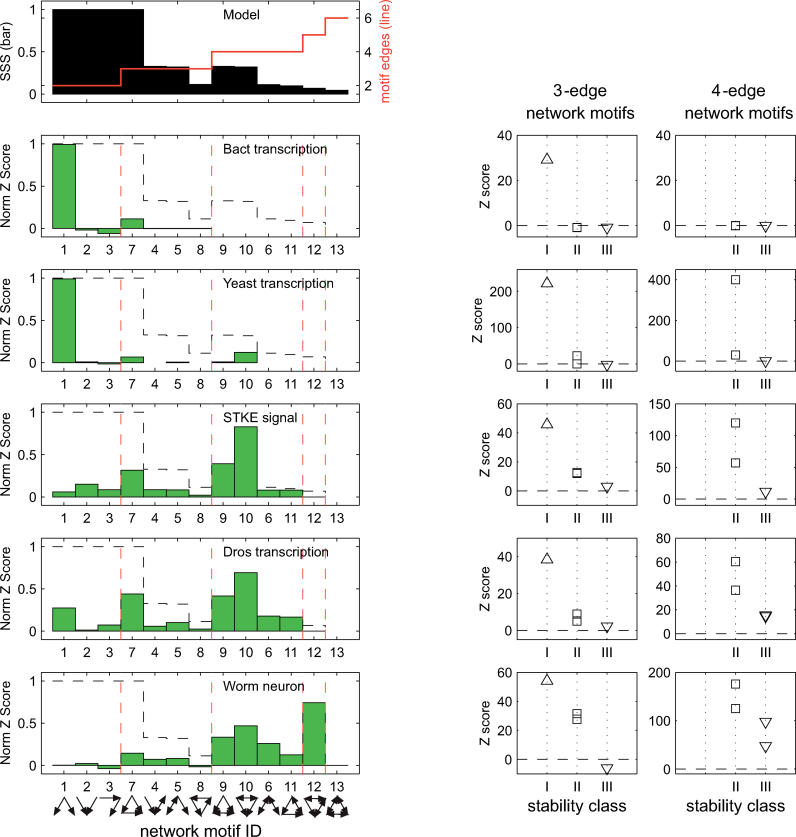
Distribution of Normalized Z Scores of Three-Node Network Motifs (Left), and Z Scores Classified by Stability within Specific Density Groups (Right) (Top panel, left) All 13 network motifs are sorted on the *x*-axis first, according to increasing number of edges (solid red line). For a given number of edges, they are then sorted from high to low SSS (black bars). (Bottom five panels, left) Normalized Z scores (green bars) for all 13 motifs of the transcriptional networks of *E. coli* and *S. cerevisiae*, STKE network, *D. melanogaster* developmental transcriptional network, and *C. elegans* neuronal network, shown with outlines of the SSS from the top panel (dotted black outline provided as a guide to the eye). Each vertical red line indicates a change in the number of motif edges, indicating boundaries of density groups. (Right) Z scores of network motifs in the three-edge and four-edge density groups are plotted in columns labeled I, II, and III, corresponding to the three stability classes, SSS = 1, SSS ≈ 0.4, and SSS < 0.2, respectively. Note that the four-edge density group does not contain any motifs with SSS = 1 since four edges among three nodes dictates at least one feedback loop.

**Figure 4:**
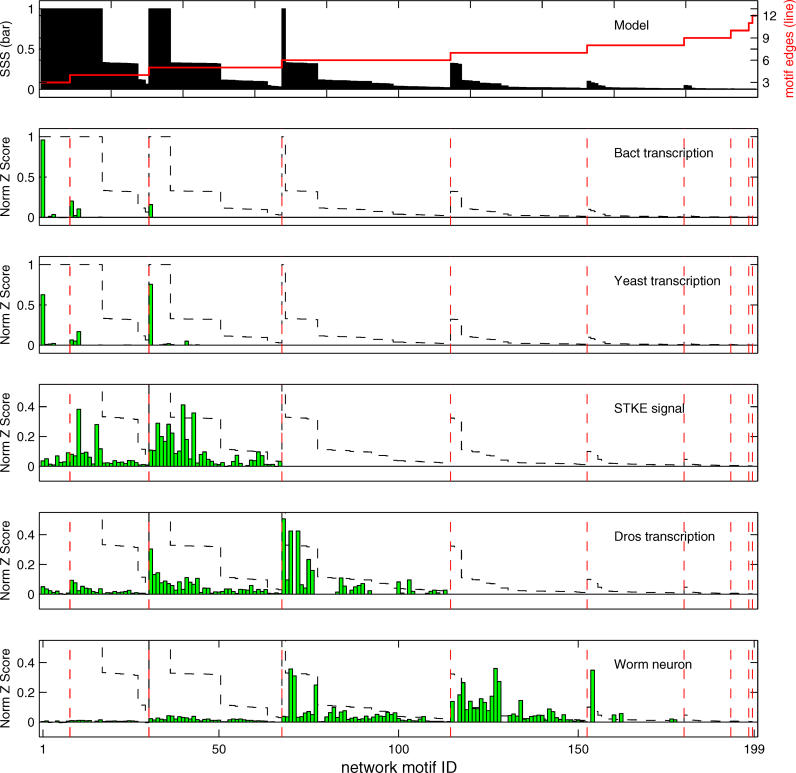
Distribution of Normalized Z Scores of Four-Node Network Motifs (Top panel) All 199 motifs are sorted on the *x*-axis first, according to increasing number of edges (solid red line). For a given number of edges, they are then sorted from high to low SSS (black bars). (Bottom five panels) Normalized Z scores (green bars) for all 199 network motifs of the indicated biological networks, shown with outlines of the SSS from the top panel (dotted black outline provided as a guide to the eye). Each vertical red line indicates a change in the number of motif edges, indicating boundaries of density groups. The composition of the four-node density groups is specified in Protocol S1.

Is the non-random character of network organization driven, at least to some extent, by the structural stability of network motifs? If this is the case, one would expect that motifs of relatively higher stability would be overrepresented compared to their relatively less stable counterparts when compared to random networks of the same size. To test this hypothesis, we generated 100 Erdös-Renyi (ER)–type random graphs with the same number of nodes and edges as each of the real networks. Lacking any organizing principle, the distribution of motifs in this type of random graph is determined by the density of edges [24]. Although there is some controversy as to whether the ER-type or scale-free random network model is a more natural representation of a network devoid of organization, we chose the former because it is the historically accepted model of a network produced by a random process of linking nodes. Also, it agrees with our intuition that power-law degree distribution is itself a level of organization of a network. However, we also performed simulations using randomized networks (which preserve degree distribution) as the null model (Figures S3 and S4). The particular choice of null model does not affect the main conclusions of the paper. We compared motifs in real and random networks by using Z scores as a metric of statistical over- or underrepresentation, as previously proposed [3,25]. A positive Z score indicates that a motif is overrepresented in the real network, whereas a negative score indicates underrepresentation in the real network compared to random graphs. The Z score profiles were normalized to unit vectors to enable comparisons of scores across different networks. Normalized Z score profiles are represented as bar graphs in Figure 3 (left panel) and Figure 4.

The top panels of Figures 3 and 4 illustrate the computational model of stability expressed as the distribution of SSS values, while the lower panels display the data for each real network. Network motifs are ordered, left to right, from low edge-density to high edge-density. Then, motifs with a given number of edges are ordered, left to right, from high to low SSS. For example, in Figure 3, motifs 1, 2, and 3 have two links and SSS = 1. They comprise a “density group” consisting of all the motifs in which three nodes are connected by two links. The next density group is comprised of motifs 4, 5, 7, and 8, which are the motifs in which three nodes are connected by three links. Within this density group, the SSS are diverse. Motif 7 has SSS = 1, motifs 4 and 5 have SSS ≈ 0.4, and motif 8 has SSS ≈ 0.2. All the real networks have some overrepresentation of motif 7, which has a higher SSS than those of motifs 4, 5, and 8. Next, some networks have a lesser overrepresentation for motifs 4 and 5, members of the moderately stable class. The least structurally stable circuit, motif 8, is not overrepresented in any real network. Similarly, the four-edge (three-node) density group also presents an opportunity to investigate the stability trend since more than one stability class is represented. The four-edge density group consists of two moderately stable motifs (9 and 10) and two minimally stable motifs (6 and 11). Z scores classified by stability are plotted in Figure 3 (right panel). Overrepresentation of structurally stable motifs (within each density group) argues for importance of motif stability as a dynamic property affecting network organization.

Four-node network motifs are combinatoric elaborations of three-node motifs [26]. The four-node significance profiles (Figure 4) capture a richer representation of the local connectivity patterns than the three-node profiles. At this resolution both similarities and differences between the networks are immediately apparent, yet the general stability trend is the same as in the three-node analysis. The networks differ in precisely which motifs are overrepresented, but the dynamic properties of overrepresented motifs are *conserved* across all networks we analyzed. As with the three-node profiles, the network motifs with the highest Z scores also have higher SSS than the other motifs with the same number of edges.

**Figure 5:**
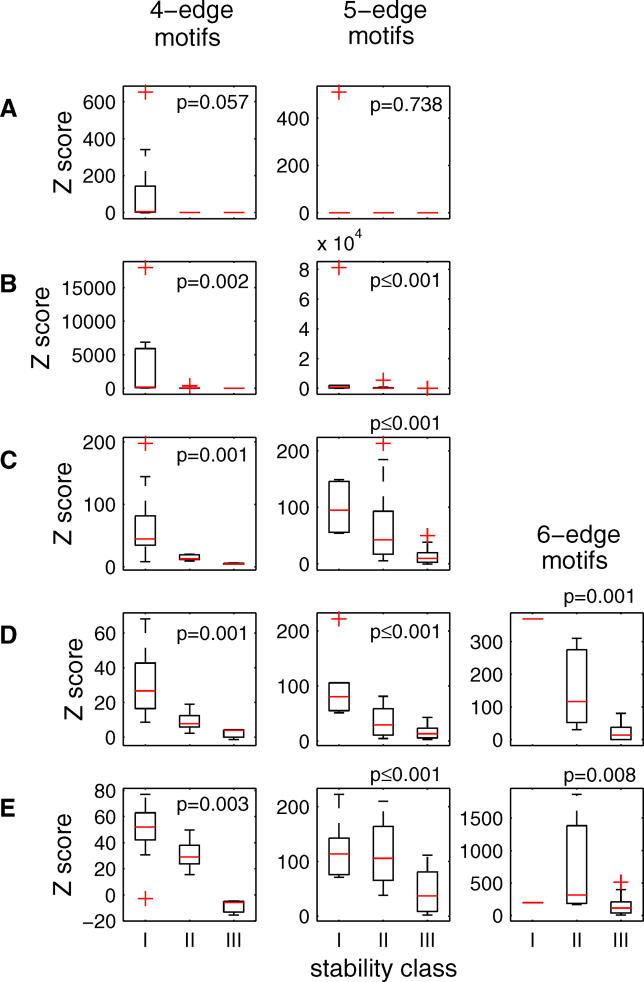
Z Scores of Four-Node Network Motifs, Classified by Stability Density groups consisting of four, five, and six-edge network motifs contain at least one motif in each stability class. Box and whisker plots indicate a difference in the average Z score between stability classes (I, II, and III correspond to SSS = 1, SSS ≈ 0.4, and SSS < 0.2, respectively). The networks examined here are the transcriptional networks of (A) *E. coli,* (B) *S. cerevisiae,* (C) STKE network, (D) *D. melanogaster* developmental transcriptional network, and (E) *C. elegans* neuronal network. A box and whisker plot is interpreted as follows: The box indicates the inner quartile range. The median Z score is indicated by a horizontal red line within the box. The whiskers extend to cover the upper and lower quartiles up to a distance of 1.5 times the inner quartile range. Red crosses indicate extreme scores (beyond the whisker length). *P*-values attached to each plot express the probability that the observed difference in Z scores between stability classes is expected by chance.

On average, the most structurally stable motifs have higher Z scores than those with lower SSS within each density group (Figure 5). For example, the *Drosophila* developmental transcription network (Figure 5D) has overrepresentation of some four, five, and six-edge network motifs. Box and whisker plots for each density group indicate that high-stability motifs (class I) have high Z scores, intermediate-stability motifs (class II) have intermediate Z scores, and low-stability motifs (class III) have low Z scores. The *p*-value attached to each plot was calculated using the Kruskal-Wallis test (one-way analysis of variance on ranks), which expresses the probability that the observed differences in Z scores between stability classes is expected by chance. In most cases, the difference in Z scores between stability classes is significant at the usual criterion of 95% confidence or better.

In addition to statistically significant differences in average Z scores among stability classes, there is similarity in stability properties among highly overrepresented network motifs. Table 1 demonstrates the preference for structural stability among the highly overrepresented network motifs in density groups consisting of four, five, and six edges (four nodes). We selected the most significant network motifs in each density group by picking Z scores in excess of one standard deviation above the mean Z score for the group. These motifs dominate the non-random organization of the network within their density group. The stability classification (I, II, or III) is indicated in parentheses next to the Z score. In all cases, the highly significant network motifs belong to classes I or II. Finally, Table 1 reports the size of each stability class, which indicates, to some extent, avoidance of low-stability motifs. For example, the six-edge density group is comprised of 47 network motifs: one class I, nine class II, and 37 class III. The *Drosophila* developmental network contains a highly overrepresented class I motif, which has the highest Z score in the density group. The remaining high Z scores correspond to class II motifs. None of the 37 low-stability motifs have high Z scores. Overall, high Z scores correspond to high SSS, but an SSS equal to unity does not always result in a high Z score, implying that structural stability may be necessary, but not sufficient, for network motif overrepresentation.

**Table 1:**
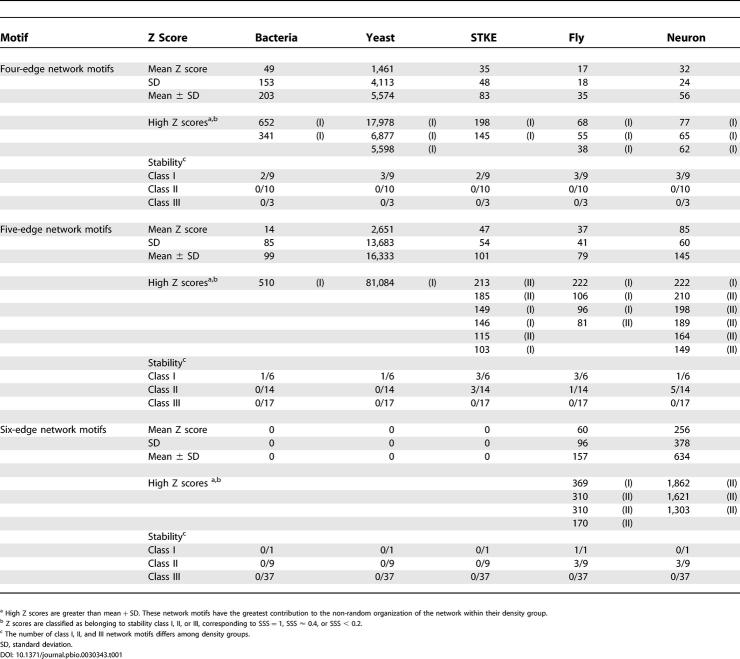
High Z Scores of Four-Node Network Motifs

In the preceding investigation, we extended a structural analysis technique (decomposition of a network into subgraphs) to a dynamical analysis. Our characterization of motif dynamics implicitly assumed that subsystems consisting of a few nodes could behave relatively autonomously despite being embedded in a larger network. Conceptually, the assumption is valid when the activity of a node does not propagate a long distance through the network, creating the situation in which small subgraphs are functionally relevant. This situation can arise when (i) only a small fraction of the nodes are activated, or (ii) only a small fraction of the potential regulatory interactions are activated. Under these conditions, the “active network” is a fragmented version of the full potential network. Note that the preceding dynamical analysis was appropriate for only a small perturbation of the activities of the nodes. Now we utilize the concept of a small perturbation to demonstrate that the active network can be a fragmented version of the full potential network, with true functional regulatory units consisting of three or four nodes.

Regulation of gene expression is dependent on the particular demands of a cell with respect to its environment. For example, many transcription factors are active only in the presence of additional signaling molecules. cAMP receptor protein, the most connected node in the bacterial transcription regulation network, is active only when bound to co-regulator cAMP, which, in turn, is present under only special circumstances, such as absence of glucose in the medium. In the absence of a cAMP signal, cAMP receptor protein is inactive, and its links are inactive. We define a context-dependent network to be the subset of the nodes and links that are activated in a certain context, such as glucose deprivation of a bacterial culture. With this principle in mind, we used high-throughput gene expression datasets to infer the context-dependent gene regulation networks, with specific contexts defined by mild environmental stress perturbations. These contexts are relevant to the preceding dynamical analysis.

We considered five different context-dependent gene regulation networks based on the genomic expression pattern of the yeast *S. cerevisiae* subjected to relatively small environmental perturbations including mild heat shock, osmotic shock, hydrogen peroxide treatment, etc. [27]. In the context of a specific environmental shock, we mapped the activated nodes (greater than 2-fold expression change) to their locations in the network. We retained all inbound links to regulated nodes, irrespective of whether or not altered mRNA expression of the upstream transcription factor was observed. We disregarded inactive links (the links that terminate on inactive nodes). Then we obtained the distribution of the size of active functional regulatory motifs by a recursive algorithm that “walked backward” through the network to discover all the upstream regulators that affect each regulated node, similar to a method previously employed [28]. In five different contexts, the mean number of nodes in a regulatory structure ranges from 3 to 3.5 (Table 2). Furthermore, all of the active regulatory motifs (three nodes, four nodes, and larger) had SSS = 1 (see Figure S5 for examples of context-dependent regulatory motifs.) Thus, in principle, the non-random character of the yeast transcriptional network could have resulted from selection acting on small network motifs that are functionally relevant in specific environmental contexts, particularly with regard to robustness to small-scale perturbations. Given appropriate small perturbation data, similar approaches could be employed to analyze the fragmentation of other complex biological networks in various functional contexts.

**Table 2:**
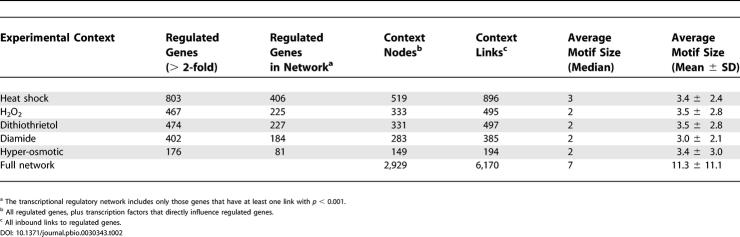
Size of Active Regulatory Motifs in the Yeast Gene Regulation Network, for Various Experimental Contexts

## Discussion

Do common “driving forces” underlie the organization of biological networks? It seems fantastic to suggest that such forces could exist, considering that the biological entities involved are as diverse as genes, enzymes, and whole cells. Nevertheless, even functionally unrelated systems may have evolved under fundamental constraints. The analysis presented here suggests that the dynamic properties of small network motifs contribute to the structural organization of biological networks. In particular, robustness of small regulatory motifs to small perturbations is highly correlated with the non-random organization of these networks. Inspection of highly overrepresented motifs in the four-node significance profile (see Figure 4 and Table 1) and statistical analysis of Z scores within and between stability classes (Figure 5) demonstrates a correlation between overrepresentation of a network motif and its SSS.

There are two separate trends in the motif significance profiles (see Figures 3 and 4). The similarity among all the examined networks is the overrepresentation of stable motifs compared to the other motifs with the same number of edges (i.e., within a density group). A separate phenomenon is that the networks differ in precisely which density groups are overrepresented, as is clearly evident in Figure 4. Previously, multiple networks have been grouped into a few families displaying remarkable similarity in the three-node motif distribution profiles [25]. At the resolution of four-node motifs (see Figure 4), bacteria and yeast transcription networks have a preference for the same network motifs. It is likely that these networks, which perform gene regulation in single-celled organisms, have evolved under similar constraints and can be considered a true “family.” However, according to the four-node motif significance profile, STKE, *Drosophila* transcription, and *C. elegans* neuron networks do not seem to form a family (as mentioned in [25] based on a three-node analysis), since they independently have preferences for different four-node network motifs. Intuitively, it makes sense that these networks do not share the same global constraints since they are built from vastly different components and have vastly different functional purposes. Initially, we hypothesized that either (i) motifs are a consequence of global constraints, as suggested by motif families, or (ii) global structure is a consequence of motifs, as suggested by constraints on robust stability of subsystems. The four-node motif significance profile (see Figure 4) demonstrates evidence for both mechanisms. Again, although there are differences in precisely which motifs are enriched in various networks, the dynamic properties of the overrepresented motifs are conserved across all networks we analyzed. Compared to network motifs with the same number of edges, the more structurally stable motifs are overrepresented. We conclude that robust stability of subsystems contributes to the global structure of the large-scale biological networks studied here.

An evolutionary argument may help explain the overrepresentation of structurally stable motifs in real networks compared to random graphs. Evolutionary pressure may select for network innovations that are structurally stable because these configurations are robust to variations in the strength of the connections. A high SSS indicates that it is likely that randomly assigned connection strengths and signs will result in a stable equilibrium, while a low SSS indicates that stability is possible although it requires precisely weighted connection strengths. Easily parameterized network designs that are predisposed to dynamical stability can be advantageous considering the evolutionary mechanisms of random mutation and natural selection. Of course, stability to small perturbations is by no means the only functional constraint on network performance and structure. For instance, in the developmental transcriptional regulation network in *Drosophila* considered here, irreversible switching of transcriptional circuits involving feedback regulation is an important determinant of irreversibility of the developmental progress, which might lead to selection of relatively unstable network motifs with feedbacks. The *C. elegans* neuron network, which strays the furthest from structural stability in our analysis, may also have functional constraints leading to overrepresentation of oscillators and memory switches. Nevertheless, the correlation between network motif overrepresentation and the SSS suggests that stability of small functional circuits may be a basic constraint common to all networks, which along with other functional requirements can significantly bias the likelihood that a given motif is selected for. Thus, one would expect to find with higher probability that some (though not all) network motifs with a high SSS would be strongly overrepresented, whereas the probability of finding overrepresented motifs with low SSS would be relatively much smaller, as suggested by Table 1.

The relationship between structural stability and overrepresentation of motifs can be illustrated using the example of three-node loops. The three-node feed-forward loop (motif 7) is an acyclic topology. Like all feed-forward architectures, it has an SSS of 1, implying that its relative abundance in a network might be high, as is indeed the case. As suggested above, structural stability is usually necessary but not sufficient for the emergence and preservation of network motifs. In the case of the feed-forward loop, previous modeling has demonstrated that this motif can produce a wealth of computational functions [5], providing functional explanations for its overrepresentation. Moreover, although all eight possible sign combinations (describing whether interactions between nodes are positive or negative) have the same SSS, only two are abundant in the transcriptional networks of *E. coli* and *S. cerevisiae* [5], providing further evidence of a particular role for this motif in these networks. On the opposite side of the SSS spectrum, the corresponding three-node feedback loop (motif 8) has a low SSS score implying low abundance, which is the case. The low SSS suggests that any possible implementation of feedback loop will be more prone to instabilities, stochastic fluctuations, and oscillations (also proposed in [25], which has indeed been demonstrated experimentally for a synthetic feedback loop network, the “repressilator” [29]). Our results suggest that low structural stability of this network motif is prohibitive for its evolutionary selection, whatever its potential benefits might be.

An important caveat of our work is that we have assumed that the network operates in isolation of other components of the overall system that may influence stability. Clearly, these example networks are a significant simplification of a considerably more complicated reality of biological regulation. For example, in assigning SSS values in transcriptional regulation networks, protein–protein interactions and other modifications of transcription factors and their targets are ignored. In this case, it is likely that these unmodeled components represent dynamics with faster time scales, and that the transcriptional network represents the slower, dominant dynamic behavior. Moreover, it is reasonable to conjecture that a network with higher SSS would be more tolerant of perturbations in the faster scale components than one with a lower SSS value. However, in some cases, these unmodeled components may exert considerable influence on the system. For example, in a recently published model of the heat-shock response in *E. coli,* considerable control is achieved by feedback loops involving mRNA and interactions with chaperones and proteases [30], none of which are found in the description of the transcriptional motifs. Nevertheless, despite the simplification that is inherent in our analysis, it is all the more interesting and surprising that we find the correlation between the structural stability of network motifs and their occurrence to be so strong. It may suggest that external control, though clearly present and significant in many cases, such as the heat-shock response, may still not be of overriding importance in others. As we increase our knowledge of the real biological networks, as well as our ability to include interactions at different levels, the analysis described here will still be applicable to their fuller description and may result in further insights about network structure and evolution.

Our characterization of motif dynamics implicitly assumed that subsystems consisting of a few nodes could behave relatively autonomously despite being embedded in a larger network. We demonstrated how the yeast gene expression network is only partially activated by mild environmental perturbations (heat shock, osmotic shock, etc.), concluding that nodes and links in large-scale networks can be interpreted as potentially active, rather than “always on.” A context-dependent network, composed of the subset of the nodes and links that are activated at a given moment, can be a fragmented version of the full potential network, where network motifs are on the scale of three or four nodes. We demonstrated context-dependence in yeast gene regulation, but we expect that, in general, it is appropriate to assume that all elements in a network are not “always on.” The more fragmented a network in various functional contexts, the more relevant our analysis of dynamic properties of network motifs.

In summary, our results suggest that robust stability of network motifs is an important determinant of biological network structure. This conclusion favors the intuitively appealing claim that biological networks need to be resistant to small-scale perturbation, including noise, and that this resistance may be structurally embedded in the network organization. While our analysis shows a strong correlation between network motif abundance and structural stability, it leaves us to speculate as to why motifs with various numbers of edges are overrepresented in different networks. Since the bacteria and yeast networks regulate gene expression in relatively simple organisms, many features provided by the presence of less stable, feedback-containing motifs may not be necessary or could potentially be detrimental. In contrast, transcriptional networks of higher organisms or non-transcriptional regulatory networks may benefit from the increased occurrence of feedback-containing motifs and more complex functions potentially provided by them. Stability to small perturbations can be important for robust network performance. Thus, it is reasonable to expect that motif distribution among diverse networks represents a balance of abundant, stable motifs and relatively rare, potentially unstable motifs, allowing greater functional flexibility coupled with predominant dynamic stability.

## Materials and Methods

### Computational model of motif stability

**Figure 6:**
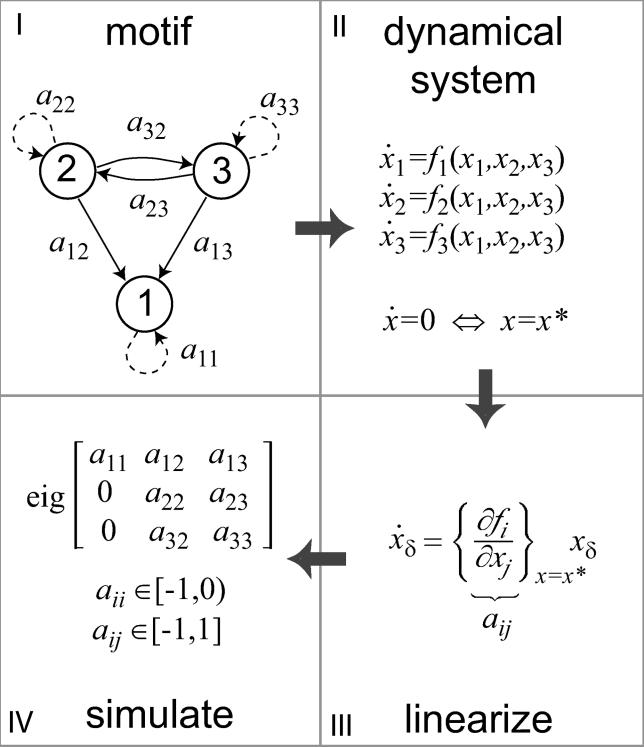
Scheme for Modeling the Local Stability of a Network Motif (I) An example network motif (10) depicted with nodes and edges labeled. The sign and weight of an edge from node *j* to node *i* is denoted *a_ij_* (solid line) consistent with the notation in the rest of the figure. Additionally, we assume that every node may have a self-interaction *a_ii_* (dotted line). (II) A motif is a graphical depiction of a dynamical system that can be modeled using (possibly nonlinear) ordinary differential equations, which are functions of the state of the nodes in the motif. (III) Assuming that the motif can achieve steady-state dynamics, the stability of the equilibrium may be analyzed by making a linear approximation of the (possibly nonlinear) functions. Linearization involves computation of the Jacobian, a matrix of partial derivatives of the functional dependencies expressed in the equations with respect to the variables. The linearized system is evaluated at the equilibrium point(s), resulting in specific values of *a_ij_*. (IV) The general form of the Jacobian matrix corresponding to this motif contains zero entries wherever one node exerts no influence on another node and non-zero elements wherever it does. An instance of a Jacobian consistent with the corresponding motif is created by sampling the non-zero terms from some distribution. Eigenvalues of the Jacobian indicate the local stability properties of the equilibrium point. Sampling many instances of structurally equivalent Jacobians allows computation of the *probability* of stability of an equilibrium point.



Given complete knowledge of the functional dependencies of the nodes, a dynamical system corresponding to a particular motif consisting of *n* interconnected nodes (Figure 6, panel I) can be represented by a system of differential equations:

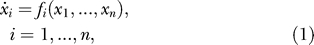



where the variable *x_i_* represents the state of the *i*th node, *f_i_* represents the combined influence of all nodes having connections to the *i*th node, and 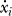 is the rate of change of *x_i_* (Figure 6, panel II). Most frequently, we do not have enough information to construct specific functions *f_i_,* which may exhibit complicated nonlinear dependencies. However, if we restrict our focus to local stability, we can alleviate the lack of functional relationships by assuming that one or more steady-states (denoted x*) exist, and examine the system behavior in a small neighborhood of these steady-states. Under these conditions, a linear approximation of the dynamics can be used (Figure 6, panel III). This approximation is accomplished by considering the evolution of small deviations of the system from the steady-state. It involves the computation of the Jacobian, a matrix of partial derivatives of the functional dependencies expressed in the equations with respect to the variables, evaluated at the equilibrium of interest.



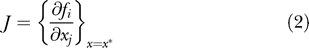



The linearized system in a small neighborhood *(δx)* of the steady-state is:



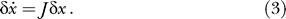



Although the precise values of the elements of the Jacobian matrix might not be known, it is clear that the matrix has zero-valued elements whenever one node exerts no influence on another node, and non-zero elements whenever it does. The sign of these elements depends on whether the influence is activating (positive) or inhibitory (negative). We note that the Jacobian can be reduced to the corresponding adjacency matrix by normalizing the entries to ones or zeros. Thus, the adjacency matrix is a particular example of a Jacobian consistent with the potentially nonlinear differential equations. Thus, in our analysis, the general form of the Jacobian *J* = {*a_ij_*} corresponding to each motif was obtained by inspection of the adjacency matrix. We also assumed that the self-connections, reflected in the diagonal terms of the Jacobian, are always negative. This assumption represents the mechanisms of constitutive degradation or inactivation of the biological entities, including gene products, phosphorylation states of signaling molecules, or depolarization states of neurons.

We analyzed the stability properties of the 13 three-node and 199 four-node motifs using root locus analysis (see Protocol S2). This technique, developed to analyze engineering control systems, allows determination of the stability of the system as a parameter is allowed to vary. Root locus and graphical methods become unwieldy for motifs containing multiple feedbacks.

Rather than calculate an exact solution for all possible three- and four-node motifs, we employed a numerical method to estimate stability under a particular probability distribution for the *a_ij_* terms. For each motif, we created 10,000 instances of the Jacobian matrix where the diagonal terms (self-interactions) were sampled from a uniform (−1,0) distribution, and the off-diagonal terms were sampled from a uniform (−1,1) distribution similar to the method described in [31]. These ranges were used since we consider linearized systems where only relative values of the *a_ij_* terms are significant. Eigenvalues were determined for each random instance of the Jacobian (Figure 6, panel IV). We defined a metric, *SSS,* as the *probability* that the dynamical system corresponding to a given motif displays a non-oscillatory, damped response to a small perturbation from a steady-state. This is equivalent to the fraction of instances in which all eigenvalues are complex numbers with negative real part and zero imaginary part. For instance, SSS = 1 indicates that non-oscillatory relaxation to a steady-state (henceforth simply termed stability) is guaranteed by the motif connectivity and does not depend on the specific parameter values that define the interactions between the variables in the corresponding dynamical system, thus making the system *structurally* stable.

### Abundance of network subgraphs

We used the Mfinder1.1 software program distributed by U. Alon's group (http://www.weizmann.ac.il/mcb/UriAlon/) to count the number of three- and four-node subgraphs in the real and random networks.

### Motif Z scores and null model

Motif abundances in the real networks were compared to those in ER- type random graphs with the same number of nodes and edges as the corresponding real network. This is a different null model than previously described [3], which utilized “randomized” networks that preserved the in- and out-degree of each node. The randomized null model is appropriate for investigating local phenomena while preserving global degree distribution. We take a completely unbiased approach, using motif abundances to study both local and global network structure simultaneously. ER random graphs were generated by first placing *n* nodes on a canvas, then adding *e* directed edges. The statistical significance of the motif abundance was evaluated by calculation of the Z score as utilized previously [25],



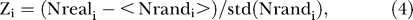



where Nreal_i_ is the abundance of the *i*th motif, and <Nrand_i_> and std(Nrand_i_) are the mean and standard deviation of its abundance in the corresponding 100 random graphs with the same number of nodes and edges as the real network. We normalized the vector of Z scores to unit length:



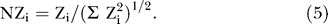



### Data sources

The *E. coli* transcription network, STKE network, *Drosophila* transcription network, and *C. elegans* neuron network described in [3,25] were obtained from U. Alon. The *S. cerevisae* transcription network was obtained from a high-throughput dataset described in [32]. We utilized the *p* < 0.001 binding interactions observed under YPD culture conditions.

## Supporting Information

Figure S1 Distribution of Normalized Z Scores of Three-Node Network Motifs Using a More Expansive Definition of SSS That Includes Damped Oscillations The SSS is represented as a stacked bar graph (top panel) in which the black portion of the bars represents the component of SSS due to monotonic decay, and the grey portion of the bars represents the component of SSS due to damped oscillations. The ordering of three-node motifs by the more expansive definition (traditional in the field of control systems engineering) produces the identical ordering of three-node motifs as conservative definitions of SSS that exclude oscillations (compare to Figure 3). Incorporating damped oscillations into the definition of stability creates a gradual continuum of stability scores. Exclusion of oscillations from the definition of stability creates a jagged landscape of SSS values, separable into classes that can also be discriminated by structural features: the number and size of feedback loops. The particular definition of SSS (including or excluding damped oscillations) does not affect the stability rank of the three-node motifs, only the raw SSS value.
(19 KB PDF).

Figure S2 Distribution of Normalized Z Scores of Four-Node Network Motifs Using a More Expansive Definition of SSS That Includes Damped Oscillations The SSS is represented as a stacked bar graph (top panel) in which the black portion of the bars represents the component of SSS due to monotonic decay, and the grey portion of the bars represents the component of SSS due to damped oscillations. The ordering of four-node motifs by the more expansive definition (traditional in the field of control systems engineering) produces a substantially similar ordering of four-node motifs as the conservative definition of SSS that excludes oscillations (compare to Figure 4). The Spearman rank correlation coefficient *(r),* which expresses the correlation between the rank of the SSS and the rank of the Z score, is indicated for selected density groups, as well as a *p*-value indicating the probability that such a correlation coefficient occurs by chance.
(168 KB PDF).

Figure S3 Distribution of Normalized Z Scores of Three-Node Network Motifs Computed Using a Degree-Constrained Null Model, Identical to the Method Previously Proposed [3,25] Overrepresented motifs (positive Z scores) tend to have higher SSS scores than other motifs with the same number of edges (compare to Figure 3). SSS does not provide insight into the occurrence of all underrepresented motifs (negative Z scores).
(19 KB PDF).

Figure S4 Distribution of Normalized Z Scores of Four-Node Network Motifs Computed Using a Degree-Constrained Null Model Identical to the Method Previously Proposed [3,25] Overrepresented motifs (positive Z scores) tend to have higher SSS scores than other motifs with the same number of edges (compare to Figure 4). SSS does not provide insight into the occurrence of all underrepresented motifs (negative Z scores).
(165 KB PDF).

Figure S5 The Regulation of Putative Yeast Gene YHR087W in Various Experimental Contexts (A) The full potential network affecting the expression of YHR087W (bottom node) was topologically sorted to reveal the causal structure. Green nodes indicate that the gene was regulated (> 2-fold change in expression) in at least one of the five environmental perturbation experiments examined here (heat shock, H_2_O_2_, dithiothrietol, diamide, hyper-osmotic shock). Green links are regulatory interactions that were inferred from the regulated genes, irrespective of whether or not the mRNA expression of the upstream transcription factor was observed. (All inbound links to a regulated node are inferred to be potentially active.)
(B) The context-dependent regulatory network in response to a mild heat shock was obtained by traversing the green links backwards from YHR087W, stopping when no more green links were encountered. The regulatory unit is larger than the average motif size of three nodes, yet dynamical stability is a robust property of the structure.
(C) The context-dependent regulatory networks for hyper-osmotic shock and diamide perturbations are a subgraph of the heat-shock regulatory structure.
(B and C) The context-dependent motifs have SSS = 1, indicating that dynamical stability is a robust property of the structure of the regulatory unit. Images generated with Cytoscape1.1 software [33].
(56 KB PDF).

Protocol S1 Four-Node Motif Dictionary ID labels match the output from mfinder1.1 motif-finding software provided by U. Alon.
(215 KB PDF).

Protocol S2 Theoretical Analysis Stability analysis using root locus.
(45 KB PDF).

## Abbreviations

ER: Erdös-Renyi
SSS: structural stability score
STKE: signal transduction knowledge environment
