# Supplementary material for: Dynamic Properties of Network Motifs Contribute to Biological Network Organization
Source: PLoS Biol. 2005 Oct 4;3(11):e343. doi: 10.1371/journal.pbio.0030343 (PMC1239925; doi:10.1371/journal.pbio.0030343)

FOUR-NODE MOTIF DICTIONARY

ID labels match the output from mfinder1.1 motif-finding software provided by Uri Alon.

Stability Class I: feed-forward

3 links

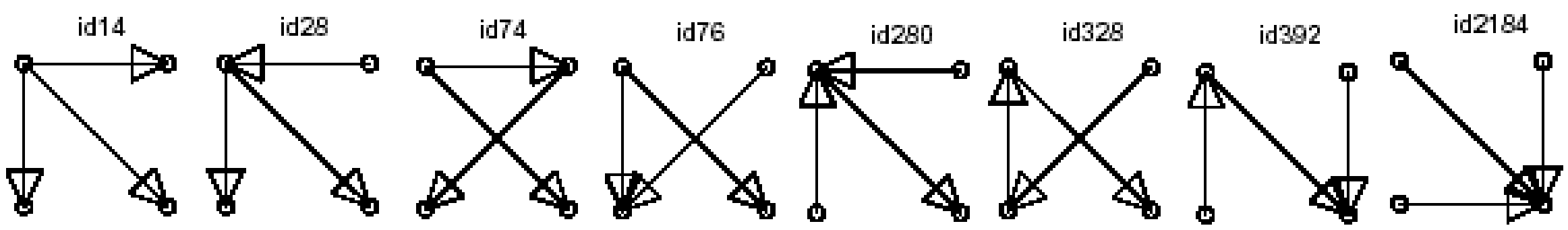

4 links

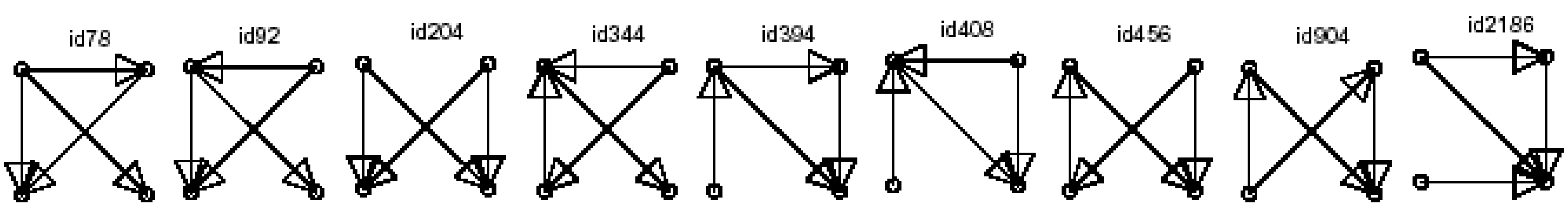

5 links

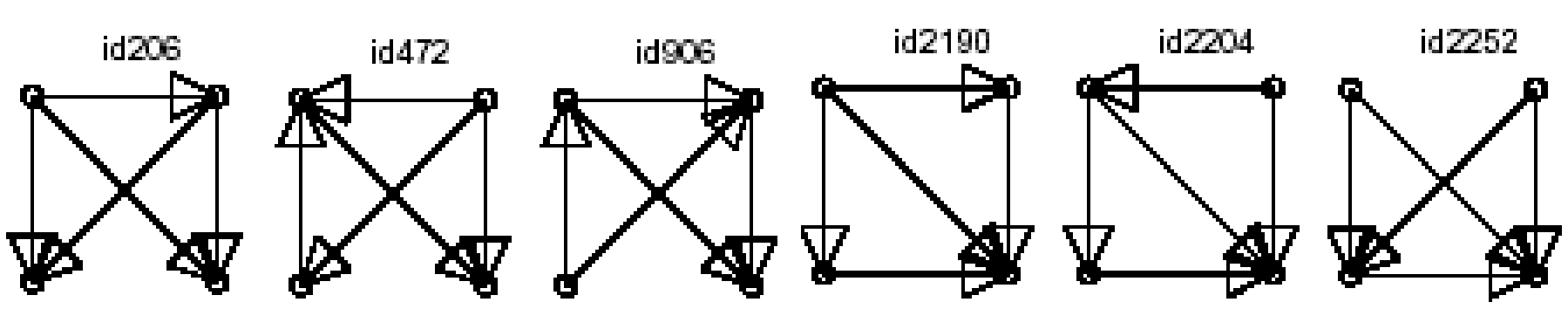

6 links

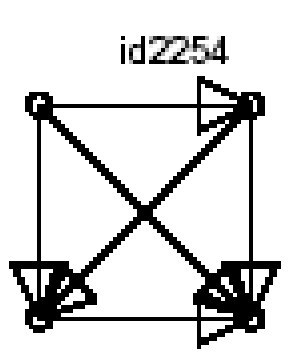

Stability Class II: one 2-node loop

4 links

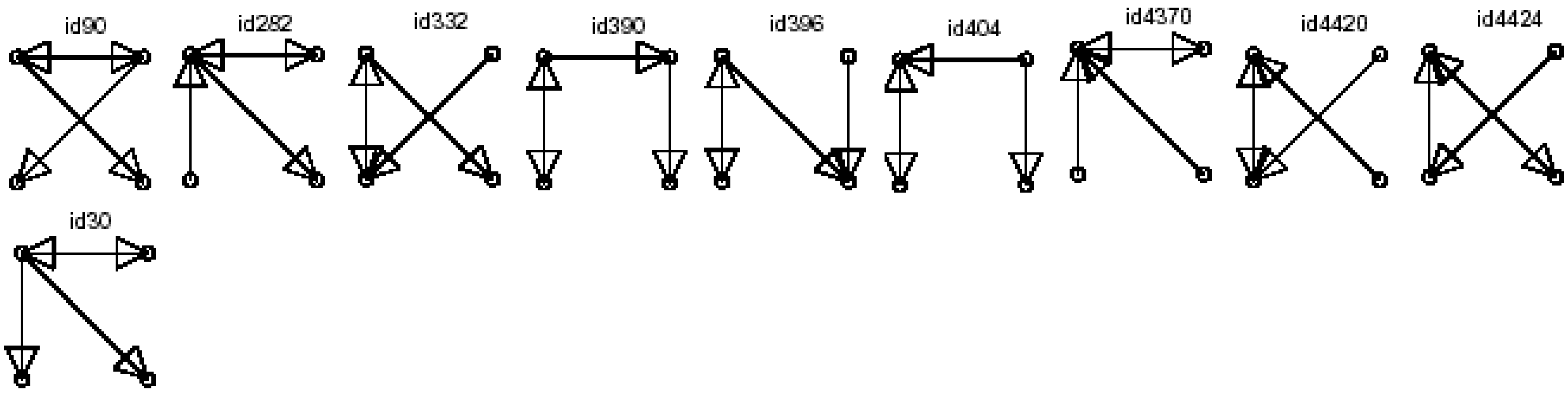

5 links

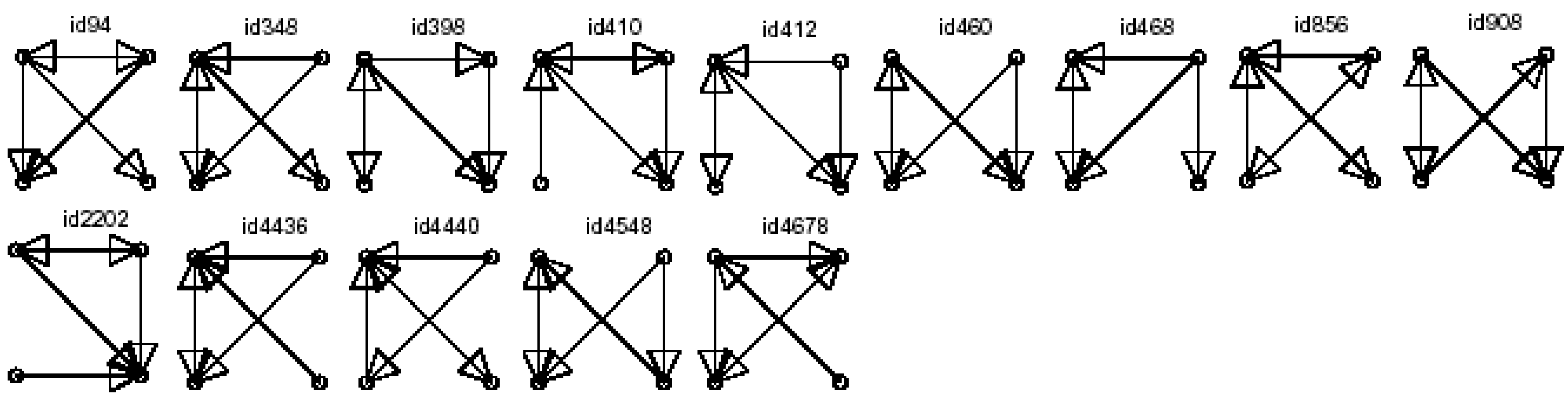

6 links

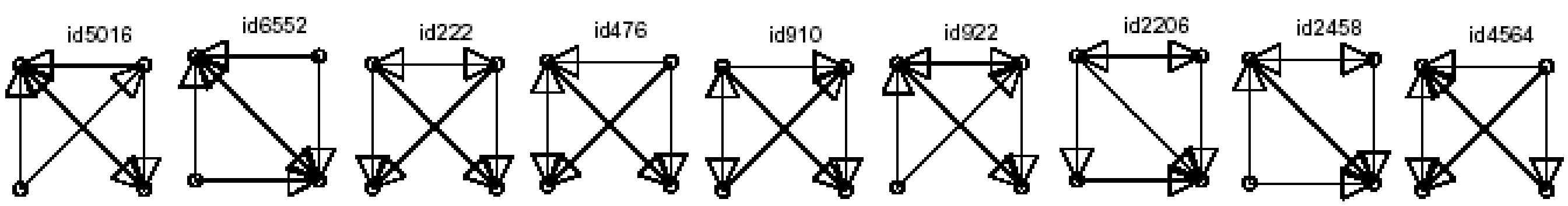

7 links

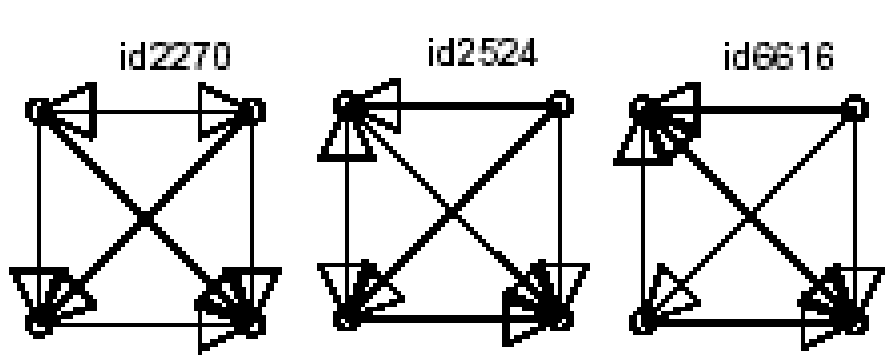

Stability Class III: more complicated loops

4 links

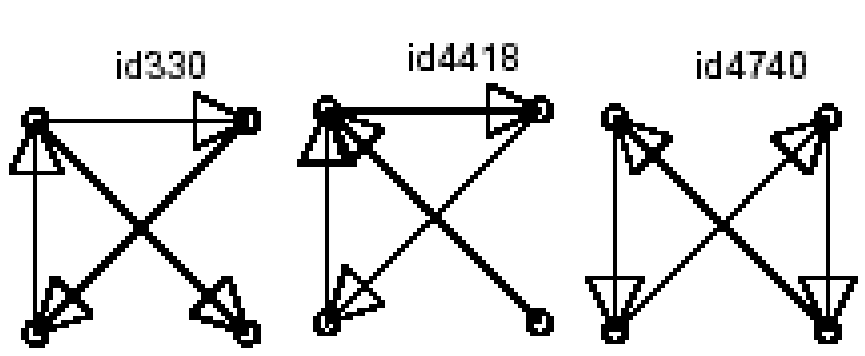

5 links

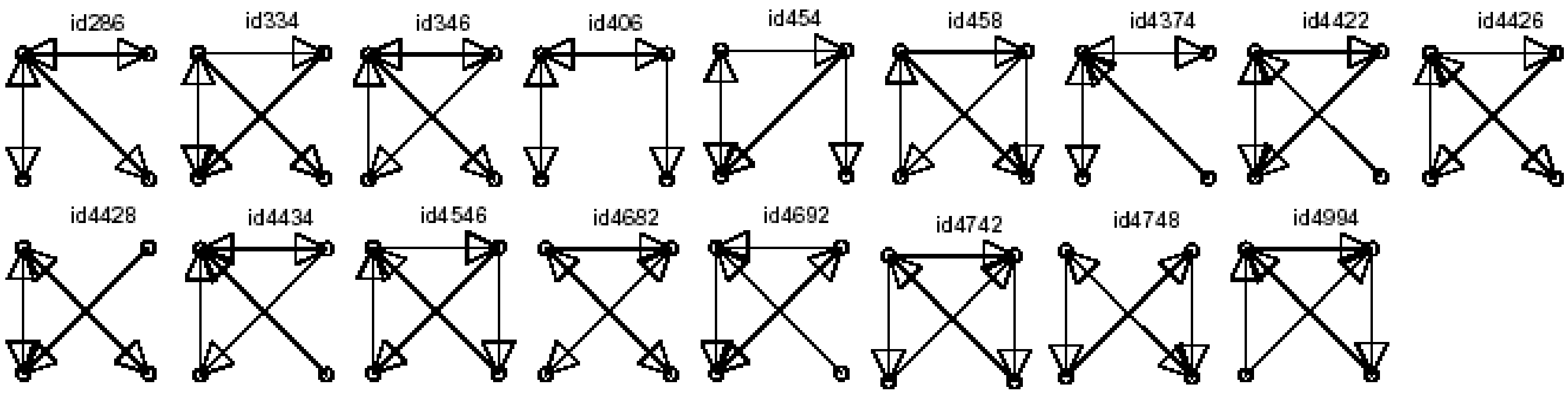

6 links

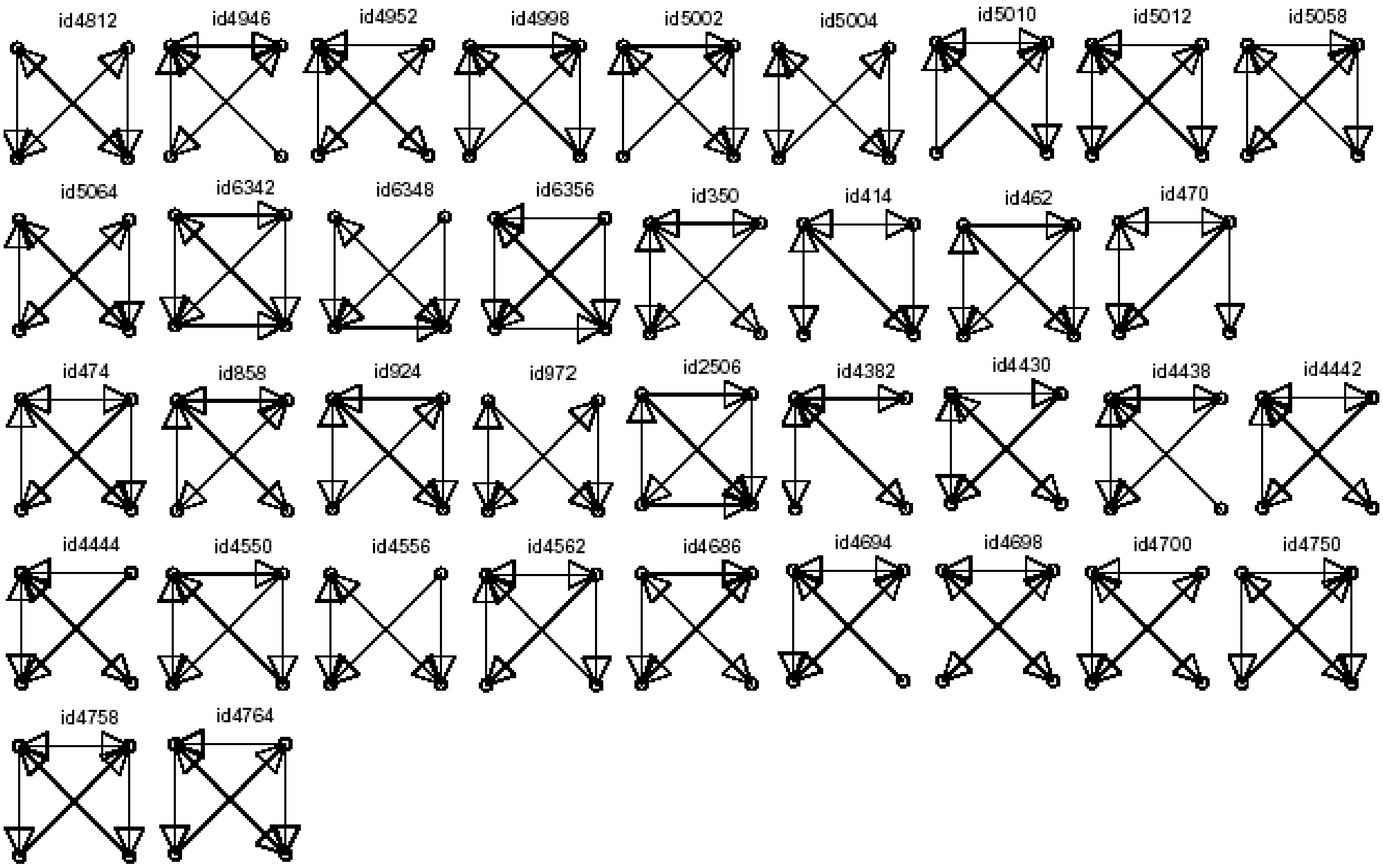

7 links

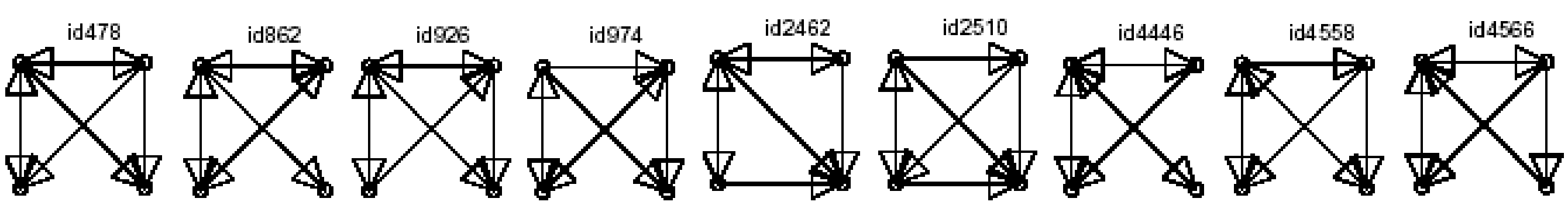

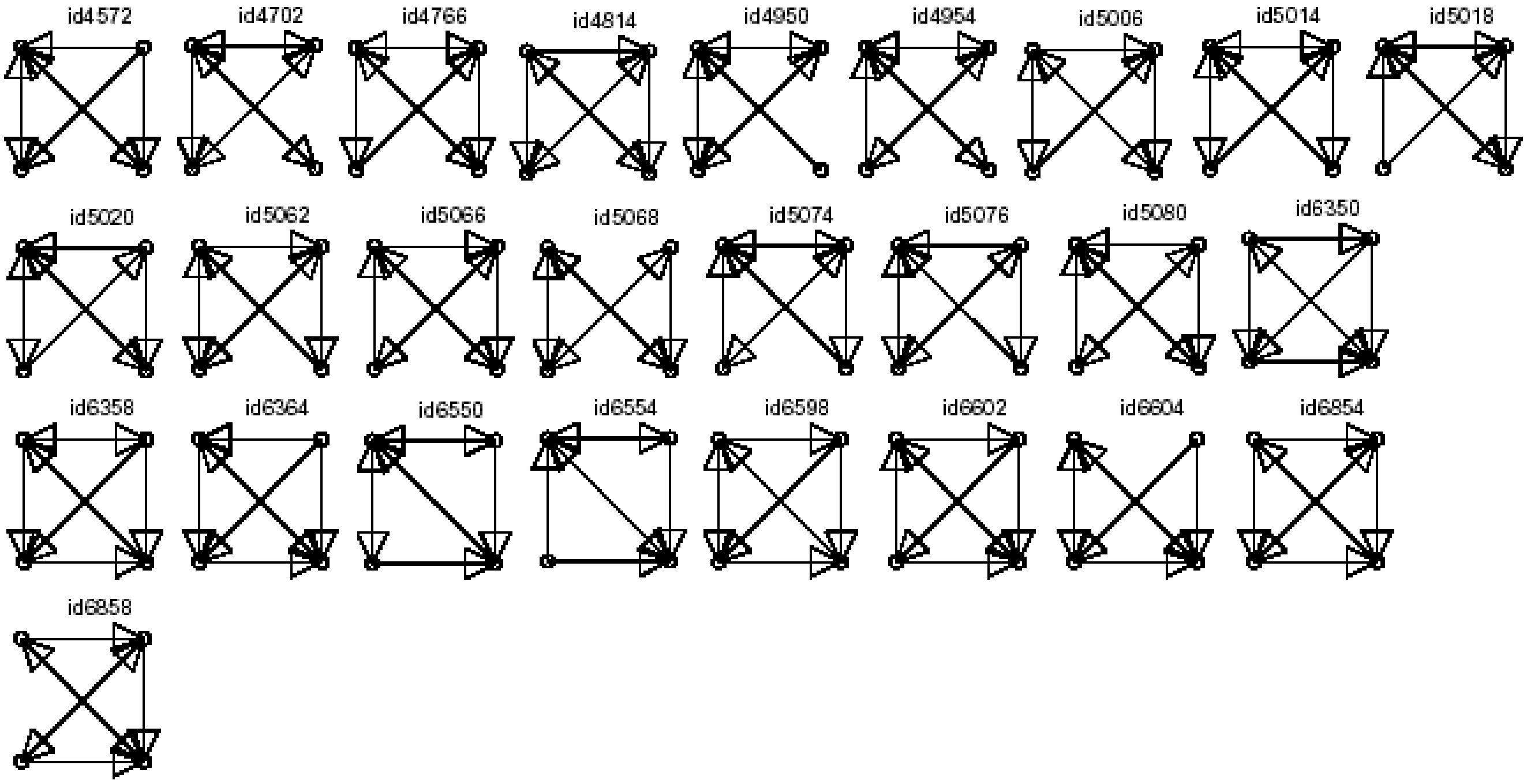

8 links

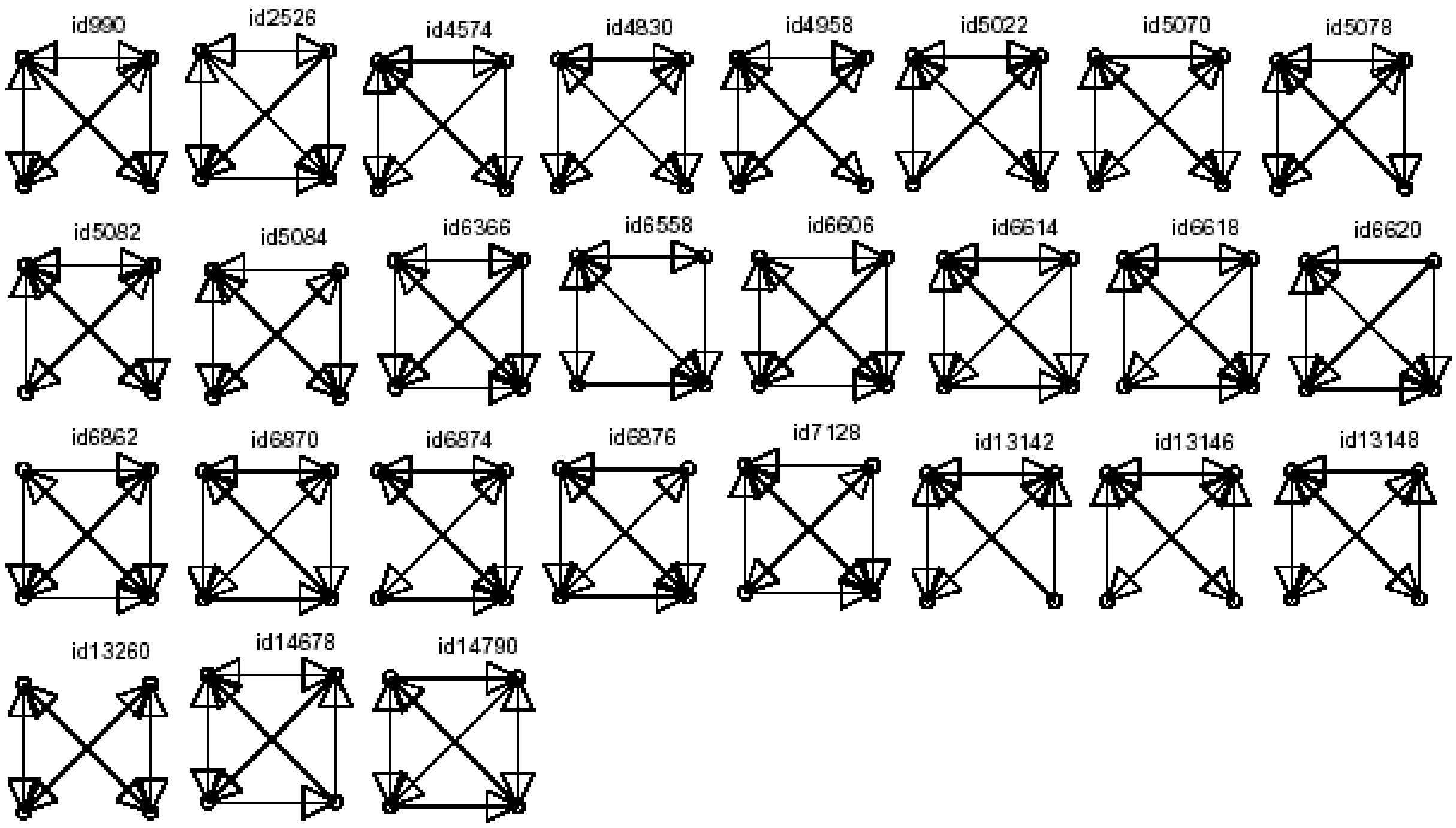

9 links

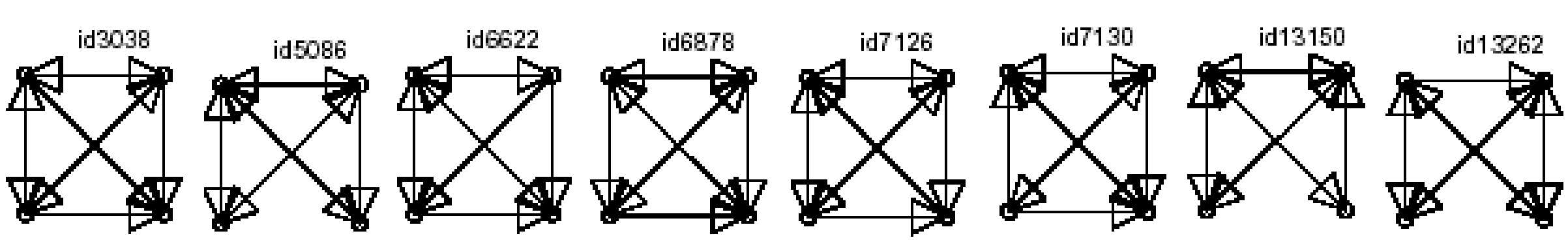

id14686

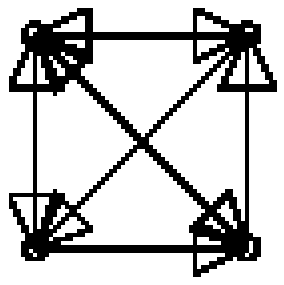

id14798

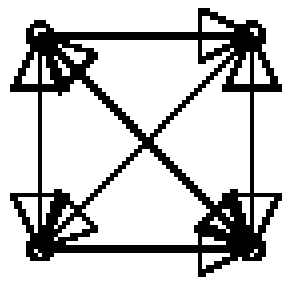

id14810

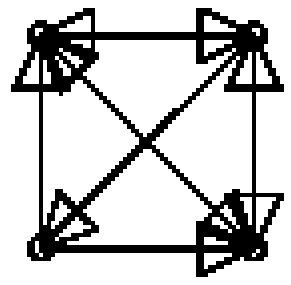

id14812

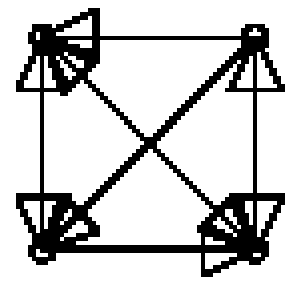

id15258

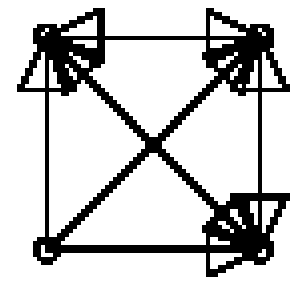

10 links

id7134

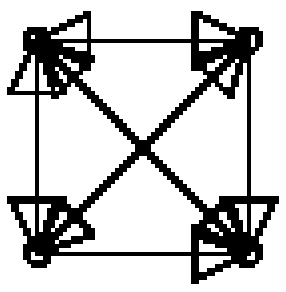

id13278

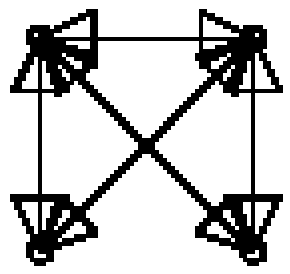

id14814

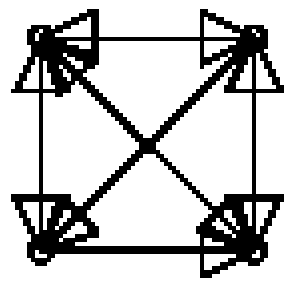

id15262

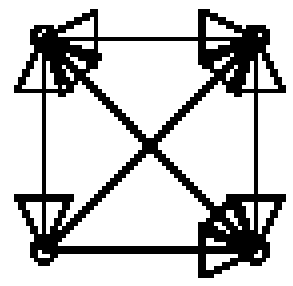

id15310

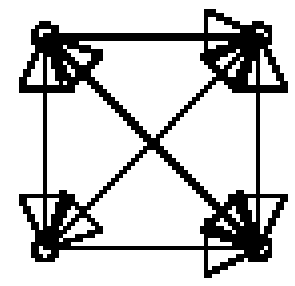

11 links

id15326

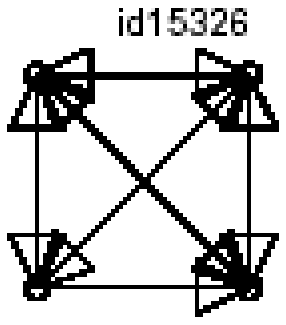

12 links

id31710

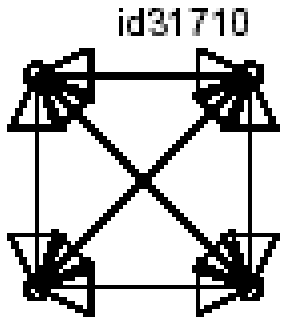

Supplement: Supplementary file 1 [file pbio.0030343.sd001.pdf]
