# Supplementary material for: Dynamic Properties of Network Motifs Contribute to Biological Network Organization
Source: PLoS Biol. 2005 Oct 4;3(11):e343. doi: 10.1371/journal.pbio.0030343 (PMC1239925; doi:10.1371/journal.pbio.0030343)
Supplement: Supplementary file 2 [file pbio.0030343.sd002.pdf]

Stability of small regulatory motifs may contribute to biological network organization

Robert Prill, Pablo A. Iglesias, Andre Levchenko

Supplementary Material

Stability analysis

The dynamical systems corresponding to a particular regulatory network motif consisting of n interconnected nodes can be represented by a system of differential equations

$$\dot{x}_i = f_i(x_1, \ldots, x_n), \qquad i = 1, \ldots, n \tag{1}$$

where the variable x_i represents the state of the ith node and f_i represents the combined influence of all nodes having connections with the ith node. The f_i may be linear or nonlinear functions. The local stability properties of the system about its (possibly multiple) equilibria can be determined using Lyapunov's indirect method (Khalil, 2002). This involves determining the location of the eigenvalues of the Jacobian matrix, $J = \{a_{ij}\} = \{\partial f_i / \partial x_j\}$, evaluated at the equilibrium of interest.

The terms a_{ij} represent the sign and strength of influence of the jth node onto the ith node. If this term is zero, the jth node does not influence the ith node at this equilibrium. Thus the Jacobian matrix serves to denote the local connectivity of the system. It can be reduced to the corresponding adjacency matrix by normalizing the a_{ij} to ones or zeros. In this study it is assumed that the self-connections for all nodes of the motifs, a_{ii} (the diagonal terms of the Jacobian matrix) are always negative. This assumption reflects the commonly observed mechanisms of constitutive degradation or inactivation of the biological entities, including gene products, phosphorylation states of signaling molecules or depolarization states of neurons. Further assumptions about the values of a_{ij} adopted in computational analysis are described below.

Open loop systems. In the following four cases,

$$J_1 = \begin{bmatrix} a_{11} & 0 & 0 \\ a_{21} & a_{22} & 0 \\ a_{31} & 0 & a_{33} \end{bmatrix}, \qquad J_2 = \begin{bmatrix} a_{11} & a_{12} & a_{13} \\ 0 & a_{22} & 0 \\ 0 & 0 & a_{33} \end{bmatrix}$$

$$J_3 = \begin{bmatrix} a_{11} & a_{12} & 0 \\ 0 & a_{22} & a_{23} \\ 0 & 0 & a_{33} \end{bmatrix}, \qquad J_7 = \begin{bmatrix} a_{11} & 0 & 0 \\ a_{21} & a_{22} & 0 \\ a_{31} & a_{32} & a_{33} \end{bmatrix}$$

the eigenvalues are just those of the diagonal terms: $\lambda(A) = \{a_{11}, a_{22}, a_{33}\}$. By assumption on the negativity of all a_{ii}, the corresponding dynamical systems are all stable, regardless of the sign or magnitude of the off-diagonal terms.

Systems with one two-node loop. In these four systems

$$J_4 = \begin{bmatrix} a_{11} & 0 & 0 \\ a_{21} & a_{22} & a_{23} \\ 0 & a_{32} & a_{33} \end{bmatrix}, \qquad J_5 = \begin{bmatrix} a_{11} & a_{12} & 0 \\ 0 & a_{22} & a_{23} \\ 0 & a_{32} & a_{33} \end{bmatrix}$$
$$J_9 = \begin{bmatrix} a_{11} & 0 & 0 \\ a_{21} & a_{22} & a_{23} \\ a_{31} & a_{32} & a_{33} \end{bmatrix}, \qquad J_{10} = \begin{bmatrix} a_{11} & a_{12} & a_{13} \\ 0 & a_{22} & a_{23} \\ 0 & a_{32} & a_{33} \end{bmatrix}$$

one of the states acts either as an input filter (in J_4 and J_9) or as an output filter (in J_5 and J_{10}). In either case, the Jacobian is block diagonal, so the spectrum of A is given by:

$$\lambda(A) = \left\{ a_{11}, \lambda\left(\begin{bmatrix} a_{22} & a_{23} \\ a_{32} & a_{33} \end{bmatrix} \right) \right\}$$

Hence, the stability of the overall system is determined by that of the 2×2 subsystem. We can think of this system as consisting of two first-order stable systems, with poles a_{22} and a_{33} and gain 1, interconnected with a feedback gain $k \equiv a_{23}a_{32}$.

In a manner complementary to the Monte Carlo analysis, we can investigate the stability of the closed-loop systems analytically using root locus arguments (Evans, 1948). This analysis tool allows determination of the stability properties of a system as a parameter is varied. Thus, rather than "sampling" the parameter space, as is the case with the Monte Carlo approach, we can consider all the possible dynamic behavior that may arise as we vary the systems' parameters over their allowable range.

In our case we determine the stability of the closed-loop system while we allow the gain k to vary. For example, if $-1 \le a_{23}, a_{32} \le 1$, it follows that $-1 \le k \le 1$. We then trace out the location of the closed-loop poles as k varies over this range. For this second-order system the closed-loop system has two poles. If $k = 0$, these closed-loop poles coincide with the open-loop poles. As k varies, these poles trace out the "branches" of the root-locus. From their location in the complex-plane, we can then determine the stability of the system.

For this second order system, if the parameters a_{23} and a_{32} are of opposite sign, so that the feedback gain is negative, ($k < 0$), the root-locus branches approach each other along the real axis, and meet at $(a_{22} + a_{33})/2$ when $a_{23}a_{32} = -4a_{22}a_{33}/(a_{22} + a_{33})^2 < 0$. For more negative values of $a_{23}a_{32}$, the branches are no longer on the real axis and approach infinity. However, they they remain in the left-half plane, implying that the closed-loop system is stable whenever $a_{23}a_{32} < 0$.

We next consider the stability of the system assuming that these parameters have the same sign, ($k \equiv a_{23}a_{32} > 0$). In this case, for increasing values of k, one branch traces the real axis starting at $\max\{a_{22}, a_{33}\}$ and enters the right-half plane whenever $a_{23}a_{32} \ge a_{22}a_{33}$. Thus, for these two cases, we can state that the system is:

$$\text{system is} \begin{cases} \text{oscillatory} & \text{if } a_{23}a_{32} \le -4a_{22}a_{33}/(a_{22}+a_{33})^2 < 0 \\ \text{stable} & \text{if } -4a_{22}a_{33}/(a_{22}+a_{33})^2 < a_{23}a_{32} < a_{22}a_{33} \\ \text{unstable} & \text{if } a_{23}a_{32} \ge a_{22}a_{33} > 0 \end{cases}$$

Single loop involving three nodes.

$$J_8 = \begin{bmatrix} a_{11} & 0 & a_{13} \\ a_{21} & a_{22} & 0 \\ 0 & a_{32} & a_{33} \end{bmatrix}$$

This can be treated as a feedback loop involving three stable, first order systems with a feedback connection with gain $k \equiv a_{13}a_{21}a_{32}$. Without loss of generality, we can assume that $a_{11} \le a_{22} \le a_{33}$.

Once again, we can use root locus analysis. In this case, there are three branches starting in the left-half plane at each of the three poles. If the feedback connection is negative ($k \equiv a_{13}a_{21}a_{32} < 0$), the two closest to the imaginary axis approach each other as before for more negative values of k. The system becomes oscillatory. However, as the magnitude of k is further increased, these two branches enter the right-half plane, so that the system is unstable. Thus, unlike system with a simple two-node feedback connection, negative feedback can destabilize the system. When $k > 0$, the branch closest to the imaginary axis remains on the real axis, but will move into the right-half plane when $k = -a_{11}a_{22}a_{33}$. The other two branches will meet. Thus, oscillatory behavior is possible, provided the feedback is not too strong, but unstable behavior is a certainty for sufficiently high values of $a_{13}a_{21}a_{32} > 0$.

Interconnections involving multiple loops. Though there are several of these, it is useful to analyze in detail the fully connected system:

$$J_{13} = \begin{bmatrix} a_{11} & a_{12} & a_{13} \\ a_{21} & a_{22} & a_{23} \\ a_{31} & a_{32} & a_{33} \end{bmatrix}$$

because the others are special cases.

This system can be treated as the feedback interconnection of the following first-order system:

$$\begin{aligned} \dot{x}_1 &= a_{11}x_1 + y \\ u &= x_1 \end{aligned}$$

with the following second order system:

$$\begin{aligned} \begin{bmatrix} \dot{x}_2 \\ \dot{x}_3 \end{bmatrix} &= \begin{bmatrix} a_{22} & a_{23} \\ a_{32} & a_{33} \end{bmatrix} \begin{bmatrix} x_2 \\ x_3 \end{bmatrix} + \begin{bmatrix} a_{21} \\ a_{31} \end{bmatrix} u \\ y &= \begin{bmatrix} a_{12} & a_{13} \end{bmatrix} \begin{bmatrix} x_2 \\ x_3 \end{bmatrix} \end{aligned}$$

The transfer function (Franklin *et al.*, 2002) from u to y is given by:

$$\begin{aligned} \frac{Y(s)}{U(s)} &= \begin{bmatrix} a_{12} & a_{13} \end{bmatrix} \begin{bmatrix} s - a_{22} & -a_{23} \\ -a_{32} & s - a_{33} \end{bmatrix}^{-1} \begin{bmatrix} a_{21} \\ a_{31} \end{bmatrix} \\ &= \frac{1}{\Delta(s)} \begin{bmatrix} a_{12} & a_{13} \end{bmatrix} \begin{bmatrix} s - a_{33} & a_{23} \\ a_{32} & s - a_{22} \end{bmatrix} \begin{bmatrix} a_{21} \\ a_{31} \end{bmatrix} \\ &= \frac{[a_{12}a_{21} + a_{13}a_{31}]s + [a_{13}a_{32}a_{21} + a_{12}a_{23}a_{31} - a_{12}a_{21}a_{33} - a_{13}a_{22}a_{31}]}{\Delta(s)} \end{aligned}$$

where

$$\Delta(s) = s^2 - (a_{22} + a_{33})s + a_{22}a_{33} - k_2, \text{ and } k_2 = a_{23}a_{32}.$$

The eigenvalues of this subsystem can be stable, oscillatory or unstable, exactly as in the analysis of the single feedback loops discussed above. The system also has a single zero, at

$$-\frac{a_{13}a_{32}a_{21} + a_{12}a_{23}a_{31} - a_{12}a_{21}a_{33} - a_{13}a_{22}a_{31}}{a_{12}a_{21} + a_{13}a_{31}}$$

which can be stable or unstable. Additionally, there will be a stable pole at a_{11}. These three poles, together with one zero, can be used to obtain a root locus analysis with gain $k \equiv a_{12}a_{21} + a_{13}a_{31}$.

The system with Jacobian

$$J_{11} = \begin{bmatrix} a_{11} & 0 & a_{13} \\ a_{21} & a_{22} & a_{23} \\ 0 & a_{32} & a_{33} \end{bmatrix}$$

is a special case of J_{13}, with $a_{12} = a_{31} = 0$. These two assumptions mean that the zero disappears. Thus, it is possible to think of this as a feedback system. Consider the interconnection of a system with two poles, with roots at the eigenvalues of $\left[\begin{smallmatrix} a_{22} & a_{23} \\ a_{32} & a_{33} \end{smallmatrix}\right]$, with a second system, with one pole at $a_{11} < 0$ and feedback gain $k \equiv a_{13}a_{21}a_{32}$. In the root locus analysis, the starting points for two of the branches are not uniquely specified. In particular, the 2×2 subsystem is of the class considered earlier. We know that these may be stable, oscillatory or unstable. If stable, then the analysis is exactly as in that of J_8. If the subsystem is oscillatory, the analysis is similar to that of J_8. If $k < 0$ the system will first continue being oscillatory, and then become unstable. If $k > 0$, the system will become unstable, but may stop being oscillatory. If the subsystem is unstable, then negative feedback will not be able to stabilize it. On the other hand, positive feedback may.

The following system

$$J_6 = \begin{bmatrix} a_{11} & a_{12} & 0 \\ a_{21} & a_{22} & a_{23} \\ 0 & a_{32} & a_{33} \end{bmatrix}$$

is also a special case of J_{13}. Since $a_{13} = a_{31} = 0$, it is easy to check that the zero is always at a_{33} and is therefore stable. Stable zeros tend to increase the overall stability of the system.

Finally,

$$J_{12} = \begin{bmatrix} a_{11} & a_{12} & a_{13} \\ a_{21} & a_{22} & a_{23} \\ 0 & a_{32} & a_{33} \end{bmatrix}$$

is almost idential to J_{13}. Unlike J_6, we can not determine the stability of the zero.

Systems involving four nodes. By breaking these systems down to simpler cases, we can say a few things. They are:

1. Open loop systems. In which the Jacobian's are all block diagonal, which are themselves triangular. Thus, the eigenvalues are the diagonal terms $\lambda(A) = \{a_{ii}\}$. Hence, these systems are always stable.

2. Involving one single-node feedback loop. These systems have block triangular adjacency matrices, and the diagonal blocks are of size two, one and one. Their stability properties are determined by the stability of the size two block.

3. Two single-loop loops. Again, the adjacency matrix is block triangular, but there are two blocks of size two on the diagonal. The analysis of the system is determined by these two blocks.

4. One single three-node loop. The adjancency matrix is block triangular with one diagonal block of size three.

5. One single four-node loop. This is straightforward to analyze, and is similar to the single three node loop.

6. Multiple, interacting loops. The way to analyze this is to break this down into feedback connection of the 3×3 subsystem

$$\begin{bmatrix} \dot{x}_1 \\ \dot{x}_2 \\ \dot{x}_3 \end{bmatrix} = \begin{bmatrix} a_{11} & a_{12} & a_{13} \\ a_{21} & a_{22} & a_{23} \\ a_{31} & a_{32} & a_{33} \end{bmatrix} \begin{bmatrix} x_1 \\ x_2 \\ x_3 \end{bmatrix} + \begin{bmatrix} a_{14} \\ a_{24} \\ a_{34} \end{bmatrix} u$$
$$y = \begin{bmatrix} a_{41} & a_{42} & a_{43} \end{bmatrix} \begin{bmatrix} x_1 \\ x_2 \\ x_3 \end{bmatrix}$$

with the first order system:

$$\dot{x}_4 = a_{44} x_4 + y$$
$$u = x_4$$

The general 3×3 subsystem has three poles and two zeros. The other subsystem's pole is stable.

References

W.R. Evans, "Graphical analysis of control systems," *Trans. AIEE*, 67, 1948, pp. 547–551.

G.F. Franklin, J.D. Powell, and A. Emami-Naeini 2002. *Feedback control of dynamic systems.* Englewood Cliffs, NJ: Prentice Hall.

H.K. Khalil 2002. *Nonlinear systems*. Englewood Cliffs, NJ: Prentice Hall.

T.I. Lee, *et al.* "Transcriptional regulatory networks in *Saccharomyces cerevisiae*," *Science*, 298, 2002, pp. 799–804.

R. Milo, *et al.* "Superfamilies of evolved and designed networks," *Science*, 303, 2004, pp. 1538–1542.

S.S. Shen-Orr, R. Milo, S. Mangan and U. Alon. "Network motifs in the transcriptional regulation network of *Escherichia coli*," *Nat. Genet.*, 31, 2002, pp. 64–68.
