# Supplementary material for: Dynamic Properties of Network Motifs Contribute to Biological Network Organization
Source: PLoS Biol. 2005 Oct 4;3(11):e343. doi: 10.1371/journal.pbio.0030343 (PMC1239925; doi:10.1371/journal.pbio.0030343)
Supplement: Supplementary file 4 [file pbio.0030343.sg002.pdf]

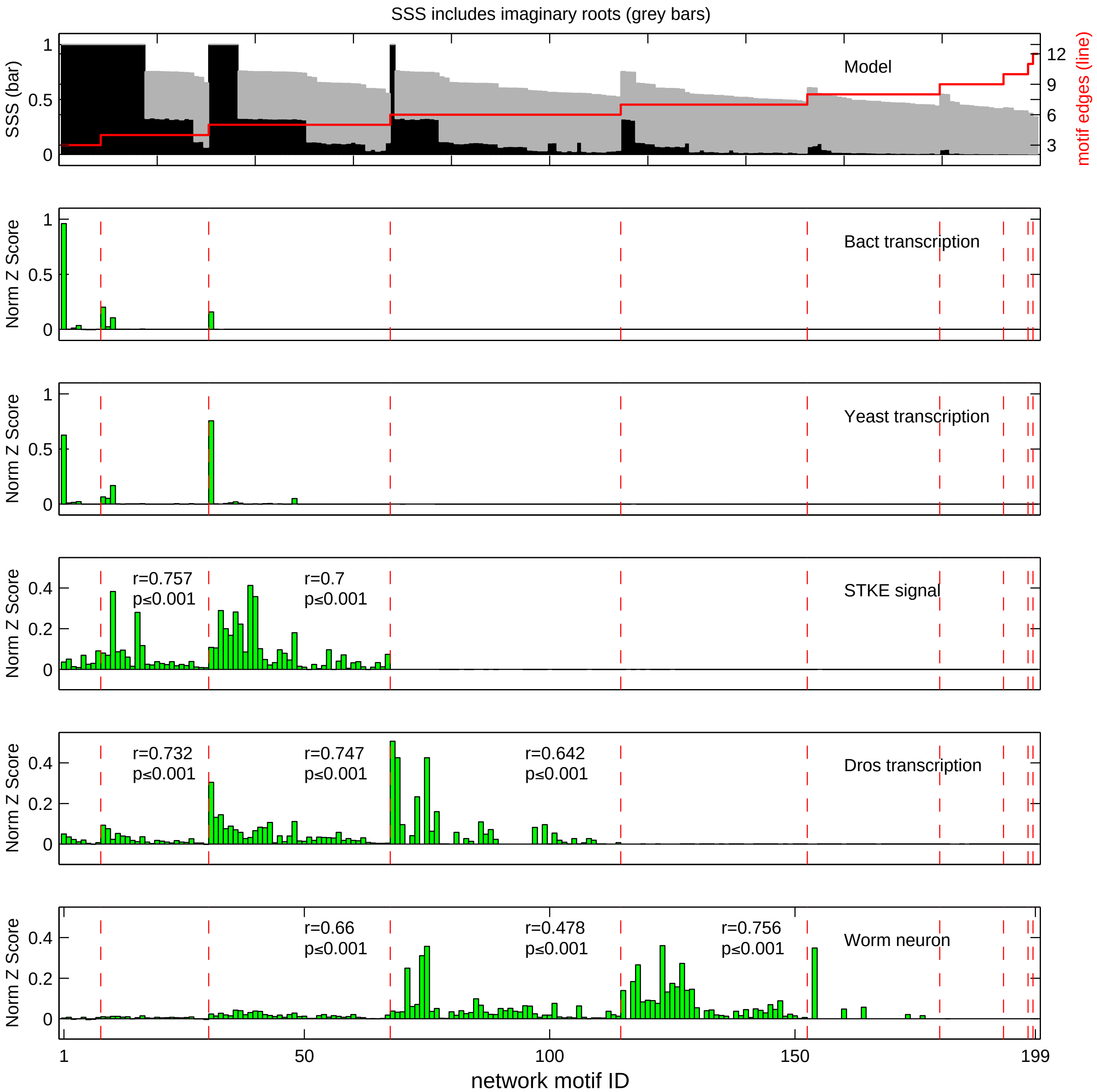

SSS includes imaginary roots (grey bars)
SSS (bar)
motif edges (line)
Model
Bact transcription
Yeast transcription
STKE signal
r=0.757
p≤0.001
r=0.7
p≤0.001
Dros transcription
r=0.732
p≤0.001
r=0.747
p≤0.001
r=0.642
p≤0.001
Worm neuron
r=0.66
p≤0.001
r=0.478
p≤0.001
r=0.756
p≤0.001
Norm Z Score
network motif ID
