# Supplementary material for: Dynamic Properties of Network Motifs Contribute to Biological Network Organization
Source: PLoS Biol. 2005 Oct 4;3(11):e343. doi: 10.1371/journal.pbio.0030343 (PMC1239925; doi:10.1371/journal.pbio.0030343)
Supplement: Supplementary file 5 [file pbio.0030343.sg003.pdf]

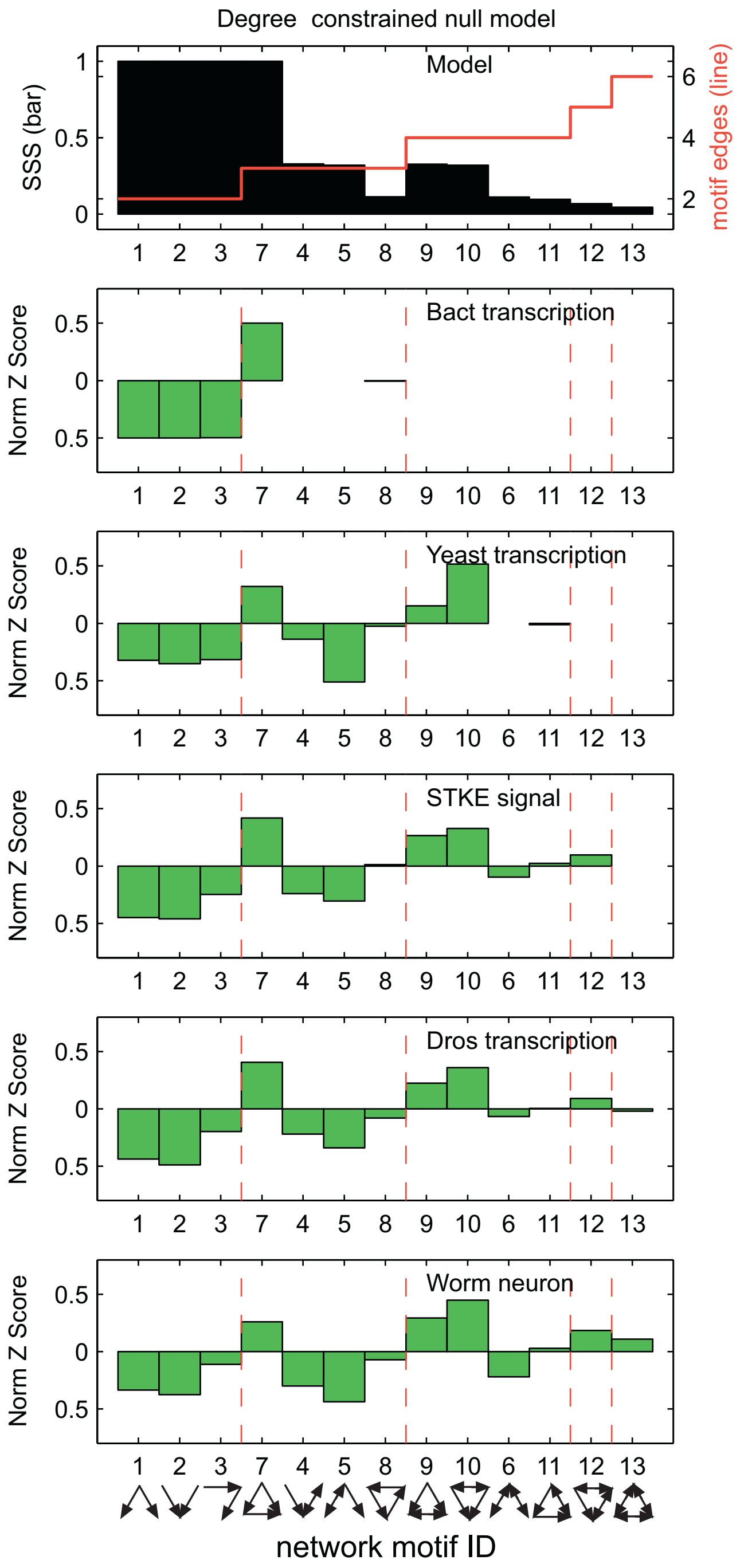
Degree constrained null model
Model
SSS (bar)
motif edges (line)
Bact transcription
Yeast transcription
STKE signal
Dros transcription
Worm neuron
Norm Z Score
1 2 3 7 4 5 8 9 10 6 11 12 13
network motif ID
