# Supplementary material for: Dynamic Properties of Network Motifs Contribute to Biological Network Organization
Source: PLoS Biol. 2005 Oct 4;3(11):e343. doi: 10.1371/journal.pbio.0030343 (PMC1239925; doi:10.1371/journal.pbio.0030343)
Supplement: Supplementary file 6 [file pbio.0030343.sg004.pdf]

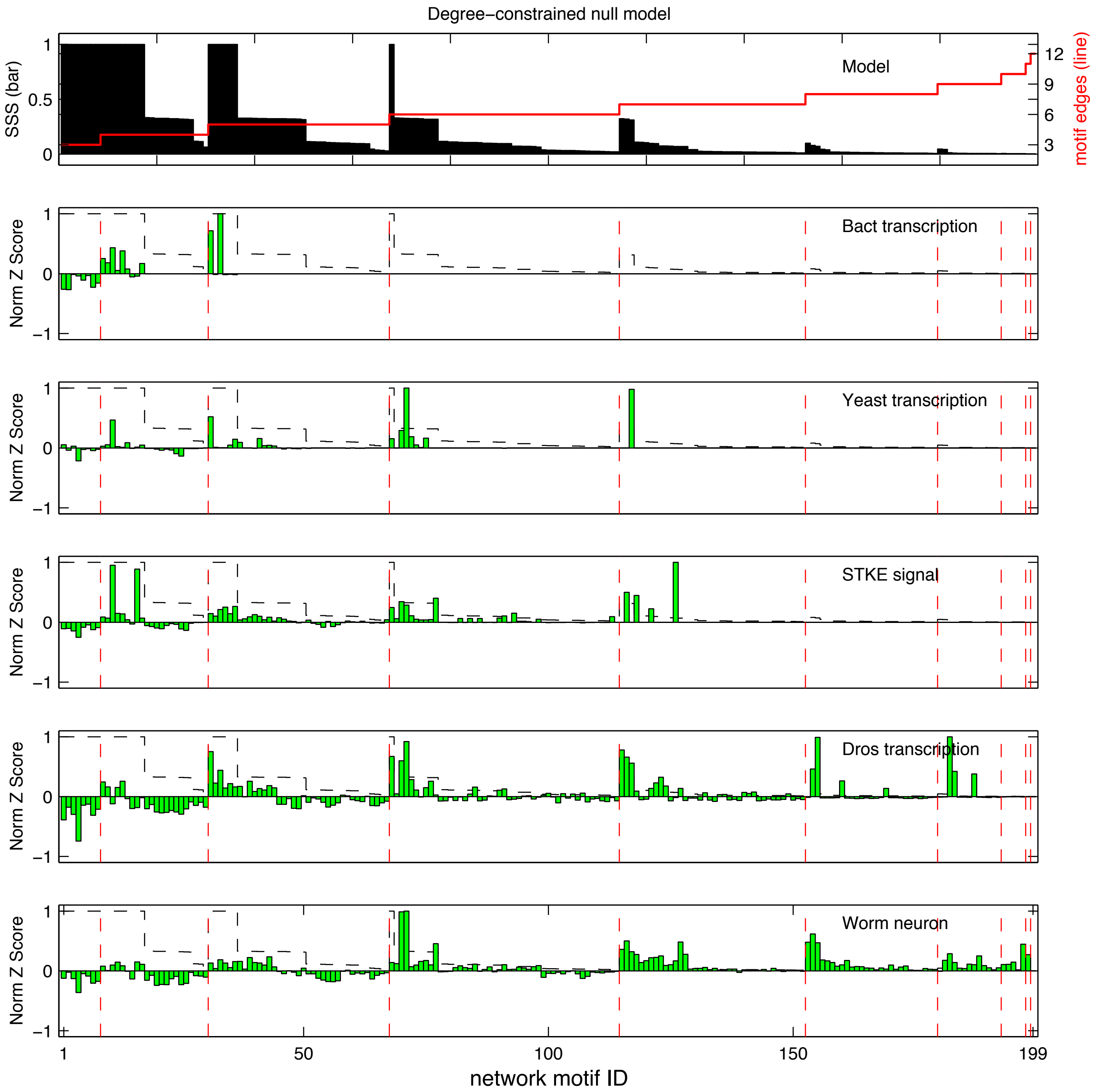
Degree−constrained null model
SSS (bar)
motif edges (line)
Model
Bact transcription
Yeast transcription
STKE signal
Dros transcription
Worm neuron
Norm Z Score
network motif ID
1
0.5
0
3
6
9
12
−1
50
100
150
199
