# Supplementary material for: Dynamic Properties of Network Motifs Contribute to Biological Network Organization
Source: PLoS Biol. 2005 Oct 4;3(11):e343. doi: 10.1371/journal.pbio.0030343 (PMC1239925; doi:10.1371/journal.pbio.0030343)
Supplement: Supplementary file 7 [file pbio.0030343.sg005.pdf]

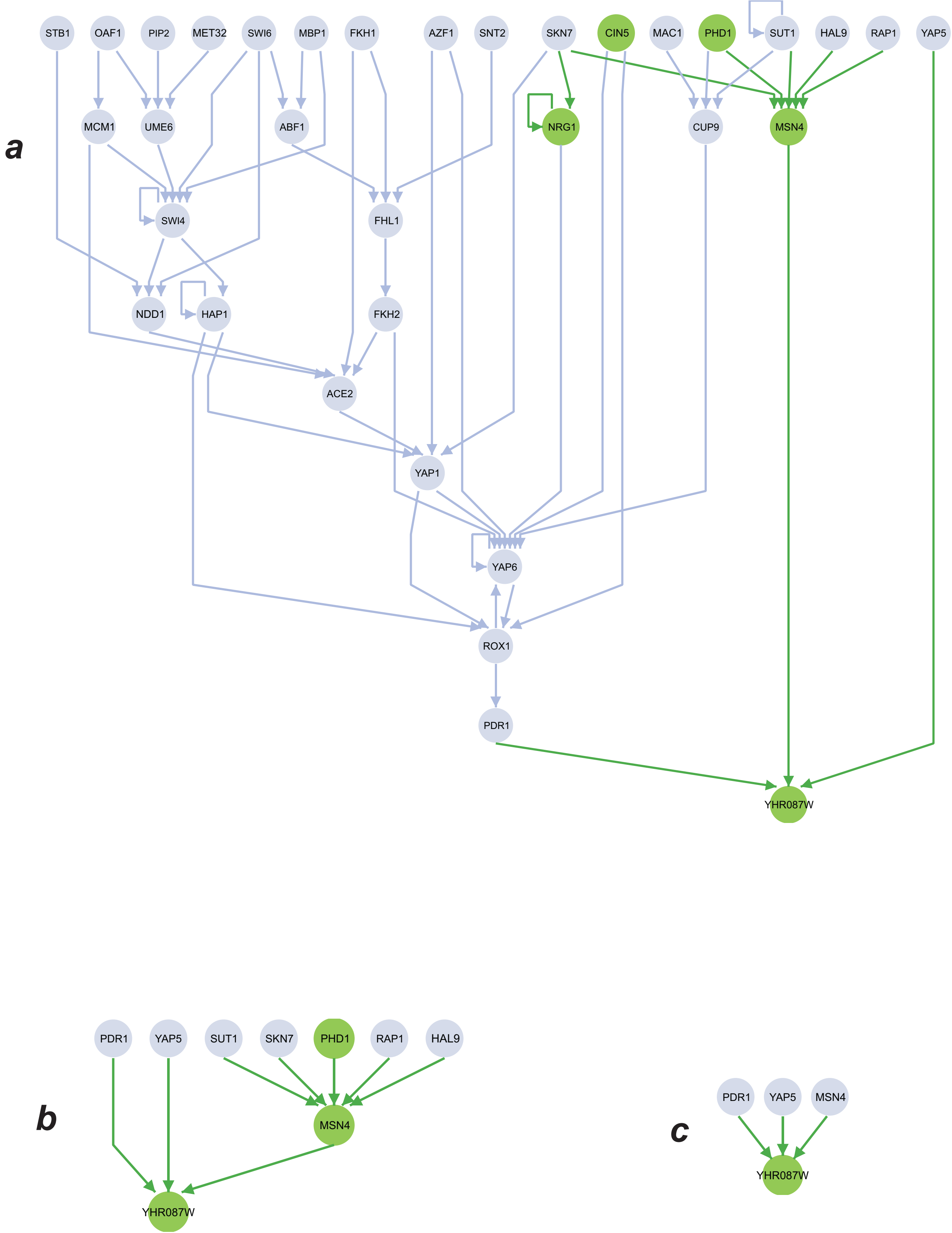
a
STB1
OAF1
PIP2
MET32
SWI6
MBP1
FKH1
AZF1
SNT2
SKN7
CIN5
MAC1
PHD1
SUT1
HAL9
RAP1
YAP5
MCM1
UME6
ABF1
NRG1
CUP9
MSN4
SWI4
FHL1
NDD1
HAP1
FKH2
ACE2
YAP1
YAP6
ROX1
PDR1
YHR087W
b
PDR1
YAP5
SUT1
SKN7
PHD1
RAP1
HAL9
MSN4
YHR087W
c
PDR1
YAP5
MSN4
YHR087W
